# Farnesyltransferase Deficiency in Cardiomyocytes Initiates Senescence and Contributes to Cardiac Fibrosis

**DOI:** 10.1002/advs.202511530

**Published:** 2026-02-26

**Authors:** Yuxiao Chen, Lian Lou, Xuan Zhang, Lin Yan, Qi Zhang, Wen Shi, Jie Ding, Xu Lin, Rong Jiang, Shuo Liu, Thida Sok, Pengli Wang, Yun Mou, Jie Han, Shenjiang Hu, Xiaogang Guo, Jian Yang

**Affiliations:** ^1^ Department of Cardiology The First Affiliated Hospital Zhejiang University School of Medicine Hangzhou China; ^2^ Department of Thoracic Surgery The First Affiliated Hospital Zhejiang University School of Medicine Hangzhou China; ^3^ Department of Cardiothoracic Surgery The Second Hospital of Jiaxing Jiaxing China

**Keywords:** FNTB, cardiac fibrosis, cellular senescence, farnesylation, LaminA

## Abstract

Cardiomyocyte senescence contributes to cardiac fibrosis, yet the molecular mechanisms remain unclear. Farnesylation is a post‐translational modification critical for cholesterol metabolism and is mediated by the farnesyltransferase beta subunit (FNTB). However, its specific role in cardiomyocyte senescence and cardiac fibrosis remains unclear. Cardiomyocyte‐specific *Fntb* knockout mice were generated to assess cardiac remodeling. RNA sequencing, DNA damage assays, and senescence markers identified molecular pathways. Mechanistic studies included nuclear envelope ultrastructure analysis, laminA assessments. Clinical relevance was assessed via human heart samples from hyperlipidemic patients. In cardiomyocyte‐specific *Fntb* knockout mice, deletion of FNTB induced progressive cardiac fibrosis that preceded hypertrophy development. Pressure overload exacerbated dysfunction in knockouts, revealing fibrosis‐dependent vulnerability. Mechanistically, loss of FNTB impaired laminA maturation, destabilized nuclear envelope integrity, and triggered DNA damage response activation, resulting in cardiomyocyte senescence. Senescent cardiomyocytes secreted elevated Tgf‐β2 and Gdf15, driving cardiac fibroblast activation. Upstream regulation studies revealed that lipid overload suppressed *Fntb* transcription via Srebf2 downregulation, recapitulated in hyperlipidemic human hearts showing reduced FNTB expression. Notably, AAV9‐mediated *Fntb* overexpression attenuated cardiac fibrosis in mice fed a high‐fat diet. Collectively, our results demonstra that lipid overload suppresses FNTB expression in cardiomyocytes. This deficiency compromises nuclear integrity, triggering a senescence program and driving cardiac fibrosis. These findings uncover a novel mechanism of lipotoxic cardiomyopathy and suggest that farnesylation warrants further investigation as a potential target to fibrotic remodeling in metabolic heart diseases.

## Graphical abstract

Lipid overload suppresses SREBF2‐mediated FNTB expression, leading to defective Lamin A maturation and nuclear envelope instability. This nuclear catastrophe triggers a pro‐fibrotic senescence program in cardiomyocytes. Notably, restoring nuclear integrity via AAV9‐based gene therapy effectively attenuates cardiac remodeling, identifying the farnesylation‐senescence axis as a promising therapeutic target for lipotoxic cardiomyopathy.

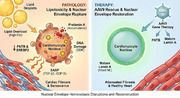

## 1 Introduction

Heart failure remains a significant global public health challenge, affecting an estimated 37.7 million individuals worldwide [1]. Cardiac fibrosis, a central pathophysiological process in heart failure progression, significantly exacerbates disease severity and prognosis. Characterized by excessive extracellular matrix deposition due to activated cardiac fibroblasts [2], fibrosis is further modulated by cardiomyocytes through paracrine signaling mechanisms that regulate fibroblast activation and function.

Cellular senescence, defined as an irreversible proliferation arrest triggered by diverse stressors, is accompanied by functional decline and apoptosis resistance. Critically, senescent cells actively release inflammatory cytokines, chemokines, proteases, and lipids through the senescence‐associated secretory phenotype (SASP), exerting profound paracrine effects on neighboring cells. Emerging evidence indicates that senescence occurs not only in mitotic cells but also in post‐mitotic cells such as cardiomyocytes [3, 4]. Senescent cardiomyocytes promote cardiac fibrosis via SASP factors like Edn3 and Tgf‐β2 [4]. Moreover, recent studies have highlighted the critical roles of autophagy [5] and post‐transcriptional regulatory mechanisms [6] in modulating cardiomyocyte senescence. However, the upstream triggers of cardiomyocyte senescence and their mechanistic links to fibrotic remodeling remain poorly understood.

Cholesterol metabolism dysregulation profoundly influences cellular senescence. Excess cholesterol accumulation accelerates senescence by compromising plasma membrane fluidity [7] and activating compartmentalized mTORC1 signaling [8]. Meanwhile, impaired cholesterol biosynthesis induces tumor cell senescence via depletion of mevalonate pathway intermediates such as ubiquinone [9]. Farnesylation, a cholesterol metabolism‐linked post‐translational modification, relies on farnesyl pyrophosphate (FPP) derived from the mevalonate pathway. Mechanistically, this process involves the covalent attachment of a 15‐carbon isoprenoid lipid to the C‐terminal CAAX motif of target proteins. This lipidation confers essential hydrophobicity, facilitating the membrane anchoring, and subcellular localization of substrates. Intriguingly, farnesol supplementation ameliorates age‐related muscle dysfunction, and neurodegeneration in mice [10, 11], whereas farnesyltransferase inhibition aggravates radiation‐induced tumor cell senescence [12]. The farnesyltransferase beta subunit (FNTB), a critical catalytic component, exhibits dynamic expression changes during cardiac development and postnatal maturation [13, 14], suggesting potential roles for farnesylation in cardiomyocyte senescence and regeneration. Nevertheless, the pathophysiological significance of cholesterol metabolism‐dependent farnesylation in cardiac senescence and remodeling remains unexplored.

In this study, employing cardiomyocyte‐specific *Fntb* knockout mouse models, we demonstrate that FNTB deficiency impairs laminA maturation, destabilizes nuclear envelope integrity, and induces cardiomyocyte senescence. These senescent cardiomyocytes secrete Tgf‐β2 and Gdf‐15, driving fibroblast activation and cardiac fibrosis. Clinical correlations further reveal reduced FNTB expression in hyperlipidemia patients’ hearts. Our findings establish a novel link between impaired farnesylation and cardiomyocyte senescence, offering mechanistic insights into cardiac remodeling associated with dyslipidemia.

## 2 Results

### 2.1 Loss of FNTB in Cardiomyocytes Promotes Cardiac Fibrosis and Diastolic Dysfunction

To interrogate the cardiac consequences of defective farnesylation, we generated tamoxifen‐inducible cardiomyocyte‐specific *Fntb* knockout (cKO) mice (Figure S1a,b), with Myh6‐MerCreMer (‐);*Fntb* ^fl/fl^ littermates as controls. RT‐qPCR validated approximately 85% *Fntb* reduction in cardiomyocytes without affecting skeletal muscle expression (Figure S1c–e).

**FIGURE 1:**
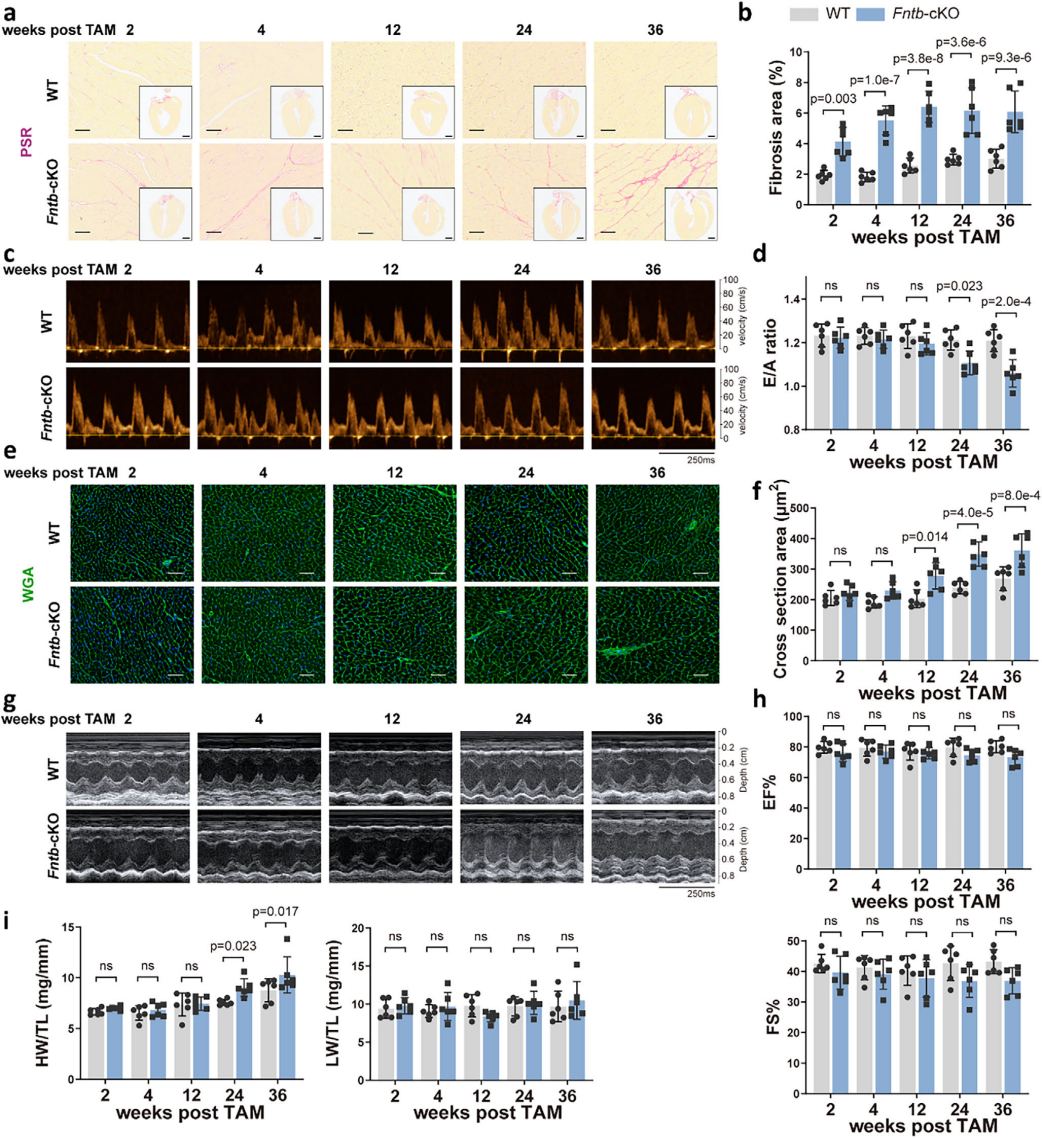
Cardiomyocyte‐specific FNTB deficiency triggers progressive cardiac remodeling and diastolic dysfunction in mice. (a,b) Representative images of Picrosirius Red (PSR) staining (a) and quantitative analysis of the fibrotic area (b) showing progressive interstitial collagen deposition in *Fntb*‐cKO hearts from 2 to 36 weeks post‐deletion (*n* = 6/group). Scale bars: 50 µm. (c,d) Representative Doppler echocardiographic flow profiles (c) and E/A ratio quantification (d) revealing diastolic dysfunction in *Fntb*‐cKO mice starting at 24 weeks (*n* = 6/group). (e,f) Representative Wheat Germ Agglutinin (WGA) staining (e) and quantification of cardiomyocyte cross‐sectional area (f) demonstrating delayed‐onset hypertrophy in *Fntb*‐cKO mice starting at 12 weeks (*n* = 6/group). Scale bars: 50 µm. (g,h) Representative M‐mode echocardiographic images (g) and quantification of ejection fraction (EF%) and fractional shortening (FS%) (h) showing preserved systolic function in *Fntb*‐cKO mice throughout the 36 weeks period (*n* = 6/group). (i) Heart weight (HW) and lung weight (LW) normalized to tibia length (TL) in *Fntb*‐cKO mice (*n* = 6/group). Data are expressed as mean ± SEM. Statistical significance was determined by two‐way ANOVA followed by Tukey's post hoc test for multiple time points. ns: not significant.

Under basal conditions, FNTB deficiency triggered a progressive cardiac remodeling program initiated by early‐onset interstitial fibrosis, which was detectable by 2 weeks post‐induction and eventually tripled in magnitude by 36 weeks (Figure 1a,b). Critically, this robust accumulation of collagen paralleled the emergence of late‐stage diastolic dysfunction, evidenced by a significant decline in the E/A ratio starting at 24 weeks (Figure 1c,d). In contrast to the rapid onset of fibrosis, cardiomyocyte hypertrophy was delayed, with increased cross‐sectional area becoming evident only at 12 weeks and significant heart weight elevation manifesting from 24 weeks onward (Figure 1e–h). Notably, while systolic function remained remarkably preserved throughout the 36 weeks observation period (Figure 1g,h), the absence of pulmonary congestion (Figure 1i) suggests that these mice maintained a compensated pathological state. Collectively, these findings demonstrate that cardiomyocyte‐specific loss of FNTB spontaneously triggers cardiac remodeling predominantly characterized by fibrosis.

**FIGURE 2:**
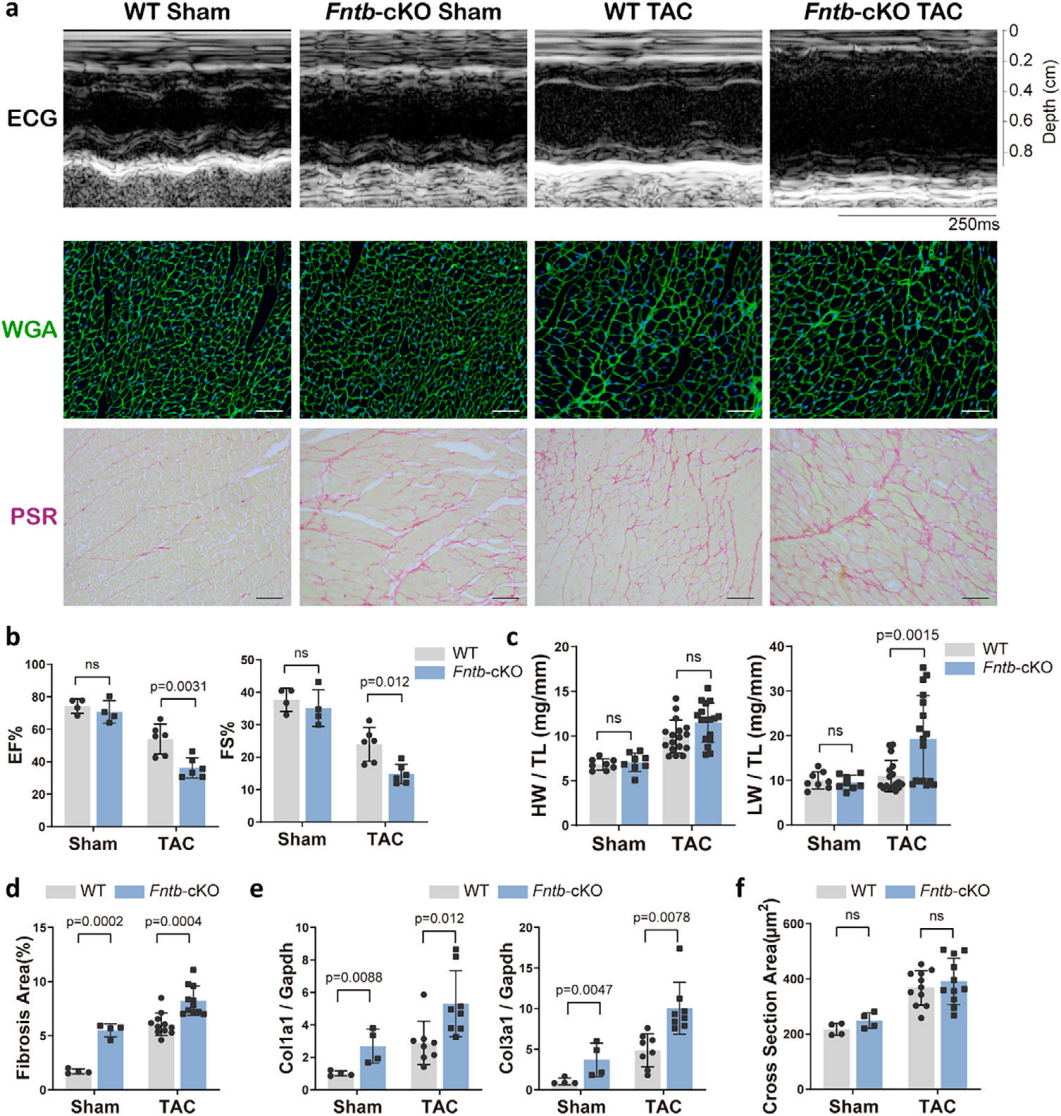
Fntb knockout exacerbates cardiac remodeling in pressure‐overload models. (a) 4 weeks after transverse aortic constriction (TAC), electrocardiogram (ECG) (top) reveals systolic function in *Fntb*‐cKO mice, with WGA (middle) staining showing cardiomyocyte hypertrophy and PSR (bottom) staining indicating interstitial fibrosis, scale bar: 50 µm; (b) Quantitative analysis demonstrates reduced EF and FS (*n* = 4–6/group); (c) TL‐normalized HW (left) and LW (right) (*n* = 8–17/group); (d) PSR staining quantification confirms elevated interstitial fibrosis in *Fntb*‐cKO hearts after TAC (*n* = 4–11/group); (e) RT‐qPCR analysis reveals upregulated *Col1a1* and *Col3a1* mRNA levels in *Fntb*‐cKO cardiac tissues (*n* = 4–8/group); (f) Cardiomyocyte cross‐sectional area analysis from WGA staining show enlarged cardiomyocyte in *Fntb*‐cKO mice (*n* = 4–11/group). Data expressed as mean ± SEM; Sham: sham‐operated controls; ns: not significant; all multi‐group comparisons analyzed by two‐way ANOVA with Tukey‐Kramer post hoc test.

Next, to probe the role of farnesylation in pathologic stress, we exposed 8‐weeks‐old *Fntb*‐cKO mice and WT littermates to transverse aortic constriction (TAC). While comparable degrees of cardiomyocyte hypertrophy were observed in both genotypes following TAC, *Fntb*‐cKO mice demonstrated accelerated functional deterioration, as evidenced by significantly impaired ejection fraction (EF) (Figure 2a,b) and prominent pulmonary congestion relative to WT controls (Figure 2c). It is suggested that the amplified dysfunction in *Fntb*‐cKO mice may arise predominantly from exacerbated fibrosis rather than hypertrophic responses. Indeed, fibrotic remodeling was significantly aggravated in cKO mice following TAC, manifesting as elevated interstitial collagen deposition and increased transcriptional expression of *col1a1* and *col3a1* genes compared with WT counterparts (Figure 2d,e). And cross‐sectional area measurements revealed no significant differences between TAC‐operated *Fntb*‐cKO and WT mice (Figure 2f).

To rule out potential confounding effects of the Myh6‐MerCreMer recombinase itself on cardiac remodeling, Myh6‐MerCreMer; *Fntb* ^wt/wt^ mice were treated with tamoxifen and monitored for 2 weeks. No pathological alterations in cardiac structure or function were observed in these control animals (Figure S2).

In summary, our findings demonstrate that the absence of cardiomyocyte FNTB induces prominent cardiac fibrosis at baseline, and significantly exacerbates cardiac dysfunction and fibrosis under pressure overload, independently of pronounced cardiomyocyte hypertrophy.

### 2.2 FNTB Deletion Triggers DNA Damage Response (DDR) and Cellular Senescence

**FIGURE 3:**
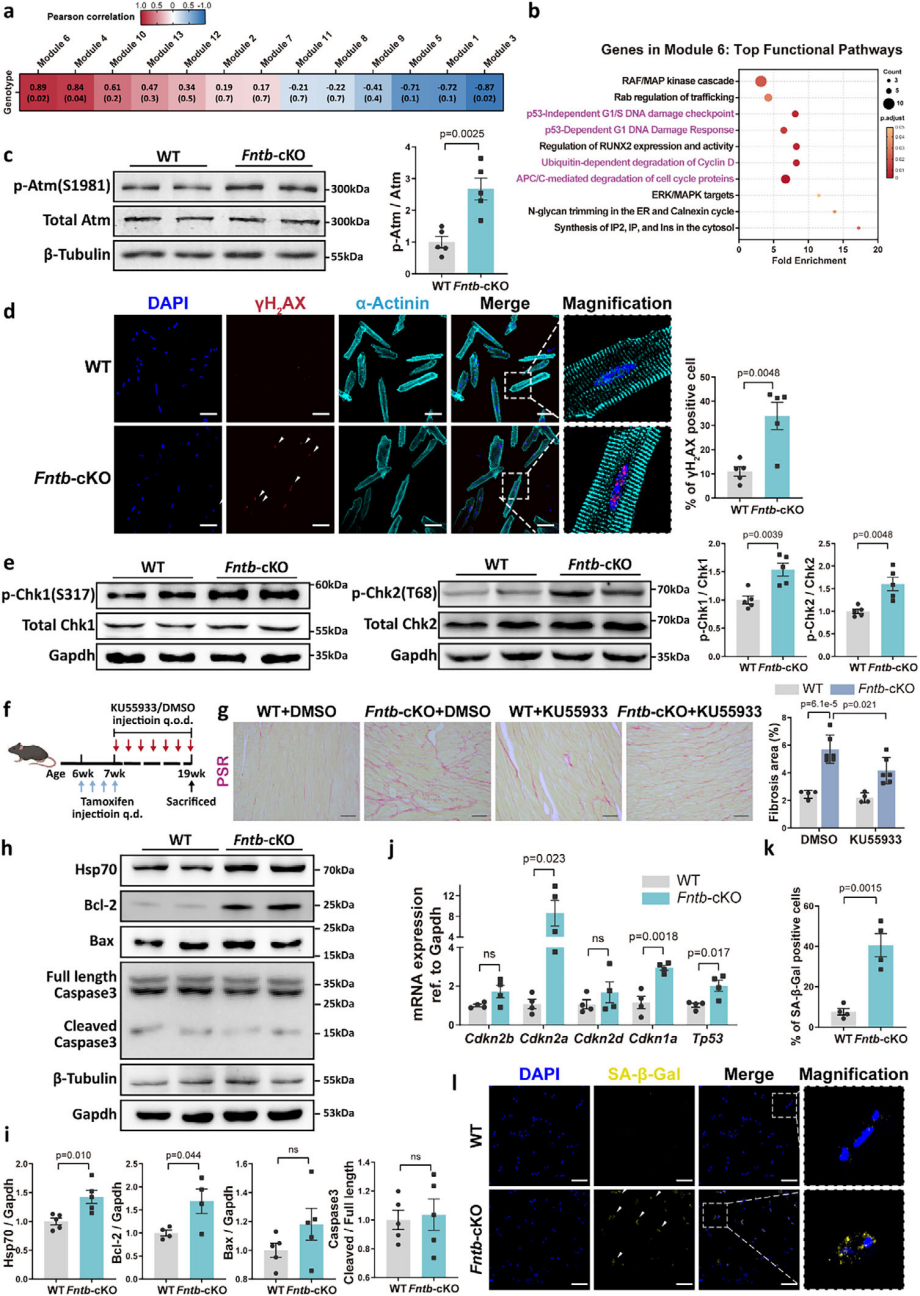
Fntb Knockout Triggers DNA Damage Response (DDR) and Cellular Senescence in Cardiomyocytes. (a) Transcriptomic profiling of adult mouse primary cardiomyocytes (AMCMs) isolated from *Fntb*‐cKO mice 2 weeks post‐tamoxifen induction. Weighted gene co‐expression network analysis (WGCNA) identified 13 gene modules hierarchically clustered by size. Module‐trait correlations are quantified by Pearson coefficients (top values) and corresponding p‐values (bottom values); (b) Top 10 enriched pathways in Module 6 ranked by fold enrichment. Circle size indicates gene count per pathway; color gradient reflects Adjusted p‐values. DDR pathways highlighted in purple; (c) Western blot analysis of Atm and phospho‐Atm (Ser1981) expression in *Fntb*‐cKO AMCMs (*n* = 5/group); (d) Immunofluorescence detection of γH_2_AX foci (white arrows) in AMCMs with quantitative analysis of γH_2_AX‐positive cells (*n* = 5/group), scale bar: 50 µm; (e) Western blot analysis of phospho‐Chk1 (Ser317) and phospho‐Chk2 (Thr68) levels (*n* = 5/group); (f) WT and *Fntb*‐cKO mice received intraperitoneal injections of the DDR inhibitor KU55933 (5 mg/kg) or vehicle (DMSO/saline) every 48 h for 12 weeks. (g) Interstitial fibrosis assessed by PSR staining and quantitative analysis in KU55933‐treated cohorts (*n* = 4–6/group), scale bar: 50 µm; (h,i) Representative Western blot (h) and quantitative analysis (i) of anti‐apoptotic proteins Hsp70 and Bcl‐2, along with pro‐apoptotic markers Bax and cleaved caspase‐3 (*n* = 4–6/group); (j) RT‐qPCR analysis of cyclin‐dependent kinase inhibitors in Fntb‐cKO AMCMs (*n* = 4/group); (k,l) Representative images (l) and quantification (k) of senescence‐associated β‐galactosidase (SA‐β‐Gal) activity in AMCMs at 24 weeks post‐induction (*n* = 4/group). White arrows denote SA‐β‐Gal‐positive cells. scale bar: 100 µm. All data represent mean ± SEM. ns: not significant; Multi‐group comparisons were analyzed using two‐way ANOVA with Tukey‐Kramer post hoc test. Two‐group comparisons were performed with an unpaired two‐tailed Student's *t*‐test.

To delineate the molecular pathways linking cardiomyocyte FNTB deficiency to cardiac remodeling, particularly fibrosis, we performed RNA sequencing on primary cardiomyocytes isolated from adult *Fntb*‐cKO mice at 2 weeks post‐ablation. Weighted gene co‐expression network analysis (WGCNA) revealed three phenotype‐associated modules (modules 3, 4, and 6; *p* < 0.05), with module 6 demonstrating the strongest correlation (Figure 3a; Figure S3a). Pathway enrichment analysis identified 48 significantly enriched pathways in module 6, with the top 10 enriched pathways primarily involving DNA damage response (DDR), regulation of cell cycle proteins, and the MAPK signaling cascade (Figure 3b). Notably, among the 48 pathways identified, 11 were closely related to the DDR pathway (Figure S3b). Consistent with transcriptomic predictions, FNTB‐deficient cardiomyocytes exhibited hallmark DDR activation, including increased Atm phosphorylation, elevated γH2AX foci formation, and Chk1/Chk2 activation (Figure 3c–e). This DDR signature was faithfully recapitulated in primary neonatal rat cardiomyocytes (NRCMs) following adenoviral‐mediated *Fntb* knockdown (Figure S4a–d). Pharmacological DDR inhibition with KU55933 attenuated FNTB ablation‐induced interstitial fibrosis and cardiomyocyte hypertrophy (Figure 3f,g; Figure S5). Collectively, these findings demonstrate that FNTB deficiency triggers a DDR in cardiomyocytes, which subsequently mediates key aspects of the observed cardiac remodeling phenotypes.

We next investigated the cellular fate downstream of this DDR activation. Surprisingly, despite robust DDR, *Fntb*‐cKO cardiomyocytes exhibited a striking resistance to programmed cell death, as evidenced by stable levels of Bax and cleaved Caspase‐3 (Figure 3h,i) and a negligible frequency of TUNEL‐positive nuclei (Figure S6a). Instead, we observed a paradoxical upregulation of anti‐apoptotic mediators Bcl‐2 and Hsp70 (Figure 3h,i), which buffers cells against DDR‐induced apoptosis (Figure S6d,e). These observations led us to hypothesize that DDR triggered by FNTB deletion may drive cardiomyocytes toward cellular senescence rather than apoptosis. Indeed, we found increased mRNA expression of cyclin‐dependent kinase inhibitors (CDKIs), including *Cdkn1a*, *Cdkn2a*, and *Tp53*, at 2 weeks post‐FNTB deletion (Figure 3j). Mirroring the temporal kinetics of the DDR (Figure S7a–d), the induction of these CDKIs emerged during the early phase following FNTB ablation and remained predominantly sustained across the majority of the observation period (Figure S7f). Subsequently, by 12 weeks post‐deletion, the *Fntb*‐cKO hearts underwent progressive chromatin remodeling, characterized by the accumulation of senescence‐associated heterochromatic foci (SAHF) as evidenced by a significant increase in H3K9me3‐positive nuclei (Figure S7g,h). This temporal trajectory was faithfully recapitulated in NRCMs following *Fntb* knockdown (Figure S4a–g). Additionally, SA‐β‐Gal activity in cardiomyocytes was significantly elevated at 24 weeks after gene deletion (Figure 3k,l).

Taken together, our findings, including robust DDR activation, elevated expression of anti‐apoptotic proteins, increased CDKIs levels, enhanced SA‐β‐Gal activity, and cardiomyocyte hypertrophy, collectively suggest that FNTB deficiency induces cardiomyocyte senescence.

### 2.3 FNTB Deletion Induces Fibroblast Activation Though Cardiomyocyte‐Derived Senescence‐associated Secretory Phenotype

**FIGURE 4:**
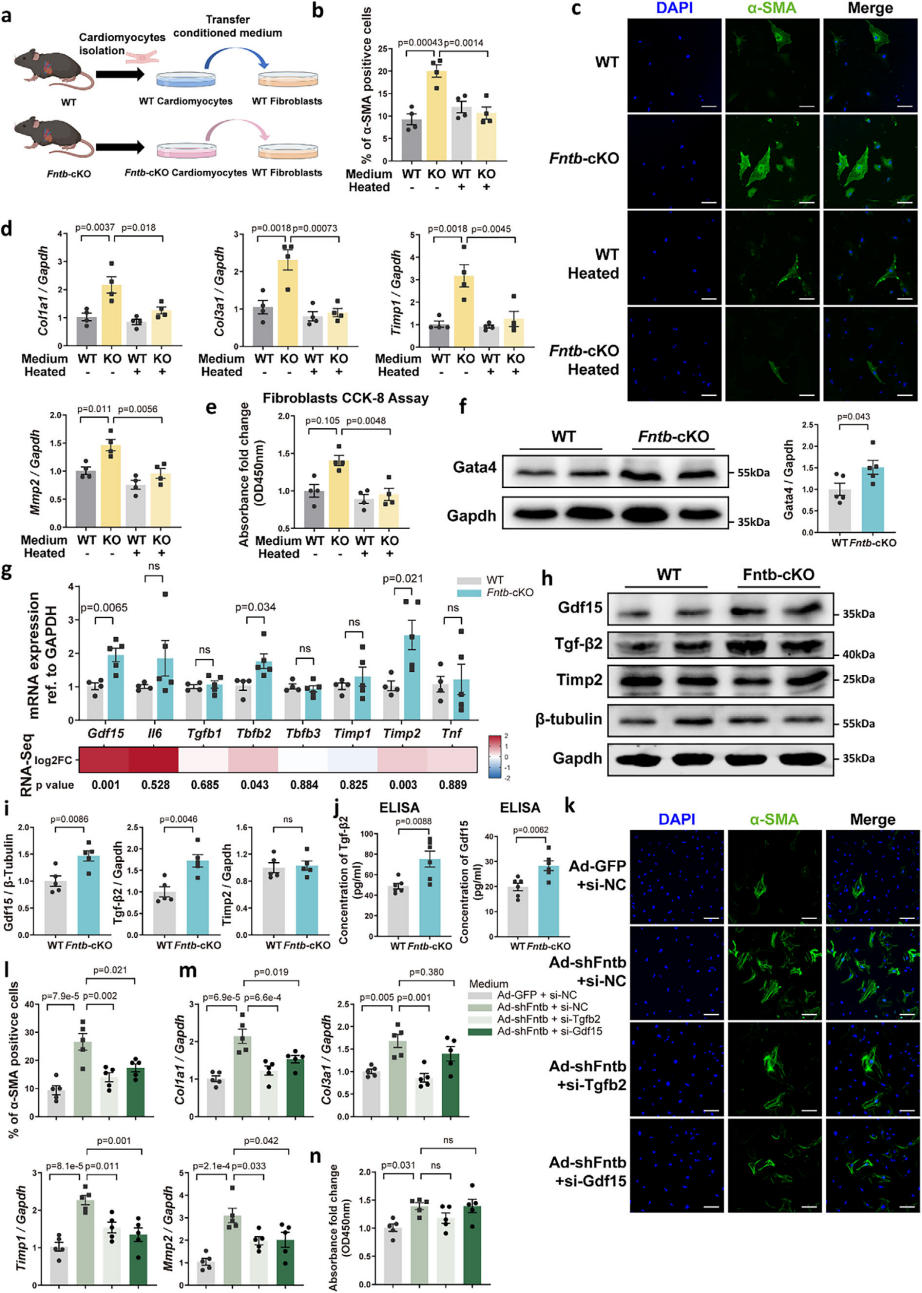
FNTB Depletion Induces Senescence‐Associated Secretory Phenotype (SASP) in Cardiomyocytes to Promote Fibroblast Activation. (a) Conditioned media transfer paradigm. Primary AMCMs from tamoxifen‐induced WT and *Fntb*‐cKO mice were cultured for 24 h. Conditioned media were collected and applied to WT cardiac fibroblasts. (b,c) Fibroblast activation was evaluated by quantifying α‐SMA‐positive cells (b, *n* = 4/group) and imaging α‐SMA expression (c), scale bar: 100 µm; (d) mRNA levels of *Col1a1*, *Col3a1*, *Mmp2*, and *Timp2* in fibroblasts treated with conditioned media were measured by RT‐qPCR (*n* = 4/group); (e) Fibroblast proliferative capacity was assessed using CCK‐8 assay under conditioned media treatment (*n* = 4/group); (f) Gata4 protein expression in *Fntb*‐cKO AMCMs was analyzed by Western blot (*n* = 5/group); (g) SASP‐related transcript levels were validated in AMCMs using RT‐qPCR (*n* = 4–5/group, upper panel), Corresponding RNA‐Sequencing data from prior analyses were displayed as a heatmap (lower panel), with color gradients reflecting expression differences between genotypes (red: upregulation in KO; blue: downregulation). (h) Protein level elevation of Gdf15, Tgf‐β2, and Timp2 in *Fntb*‐cKO AMCMs;(i) Quantitative analysis of Gdf15, Tgf‐β2, and Timp2 protein level (*n* = 5/group); (j) Secreted Gdf15 and Tgf‐β2 in cardiomyocyte conditioned media were quantified by ELISA (*n* = 6/group); (k,l) Representative immunofluorescence images (k) and quantitative analysis (l) of α‐SMA‐positive fibroblasts treated with conditioned media from Ad‐shFntb‐infected NRCMs transfected with negative control siRNA (si‐NC), si‐Tgfb2, or si‐Gdf15 (*n* = 5/group), scale bar: 100 µm; (m) mRNA levels of ECM‐related genes, including *Col1a1*, *Col3a1*, *Timp1*, and *Mmp2*, in fibroblasts under the indicated treatments were measured by RT‐qPCR (*n* = 6/group); (n) Fibroblast proliferative capacity was assessed using CCK‐8 assay (*n* = 6/group). All data represent mean ± SEM. Multi‐group comparisons were analyzed using two‐way ANOVA with Tukey‐Kramer post hoc test. Two‐group comparisons were performed with an unpaired two‐tailed Student's *t*‐test.

We next sought to define how cardiomyocyte senescence induced by FNTB deletion contributes mechanistically to cardiac fibrosis. First, conditioned media collected from cultured primary cardiomyocytes isolated from either *Fntb‐*KO or WT mice were subsequently applied to primary WT cardiac fibroblasts (Figure 4a). Remarkably, conditioned media from KO cardiomyocytes significantly enhanced cardiac fibroblast activation, proliferation, viability, and extracellular matrix secretion (Figure 4b–e).

The SASP refers to the characteristic enhancement of secretory activity observed in senescent cells. Our WGCNA analysis previously identified a significant enrichment of SASP‐related pathways in Module 6 of *Fntb*‐cKO cardiomyocytes (Figure S3b). Mechanistically, DDR‐induced SASP has been linked to the stabilization of Gata4. We first confirmed increased Gata4 protein levels in *Fntb*‐cKO AMCMs (Figure 4f). Next, given the considerable heterogeneity of SASP components across senescent cell populations, we systematically evaluated a curated panel of candidate SASP factors derived from prior studies through integrated RNA sequencing and RT‐qPCR analyses. Among these, *Tgfb2*, *Gdf15*, and *Timp2* mRNA expression levels were significantly increased in cardiomyocytes following FNTB deletion (Figure 4g), in agreement with prior findings by Anderson et al. and others [4]. Immunoblotting further confirmed elevated Tgf‐β2 and Gdf15 protein levels in *Fntb*‐cKO cardiomyocytes, while Timp2 protein levels were unchanged (Figure 4h,i). Consistently, ELISA assays demonstrated increased secretion of Tgf‐β2 and Gdf15 into the conditioned medium derived from KO cardiomyocytes (Figure 4j). This SASP signature was faithfully recapitulated in NRCMs following FNTB knockdown (Figure S4h). To confirm the functional necessity of these factors, we employed siRNA to silence Tgfb2 or Gdf15 in FNTB‐deficient NRCMs. Crucially, neutralizing either factor in the conditioned media significantly blunted its pro‐fibrotic effect, effectively attenuating fibroblast activation, and the induction of ECM‐related genes (Figure 4k–n).

To exclude the potential contribution of systemic inflammation to this process, we performed immunofluorescence staining for CD45 and F4/80 in *Fntb*‐cKO hearts. The absence of significant leukocyte or macrophage infiltration suggests that the observed fibrosis is not primarily driven by inflammatory cell recruitment, but rather by direct cardiomyocyte‐to‐fibroblast paracrine signaling (Figure S8a,b).

Collectively, our findings provide compelling evidence that FNTB deletion in cardiomyocytes induces a DDR‐dependent SASP characterized by increased production and secretion of Tgf‐β2 and Gdf15, thereby driving cardiac fibroblast activation and promoting cardiac fibrosis.

### 2.4 FNTB Deletion Impedes LaminA Maturation and Disrupts Nuclear Envelope Integrity

**FIGURE 5:**
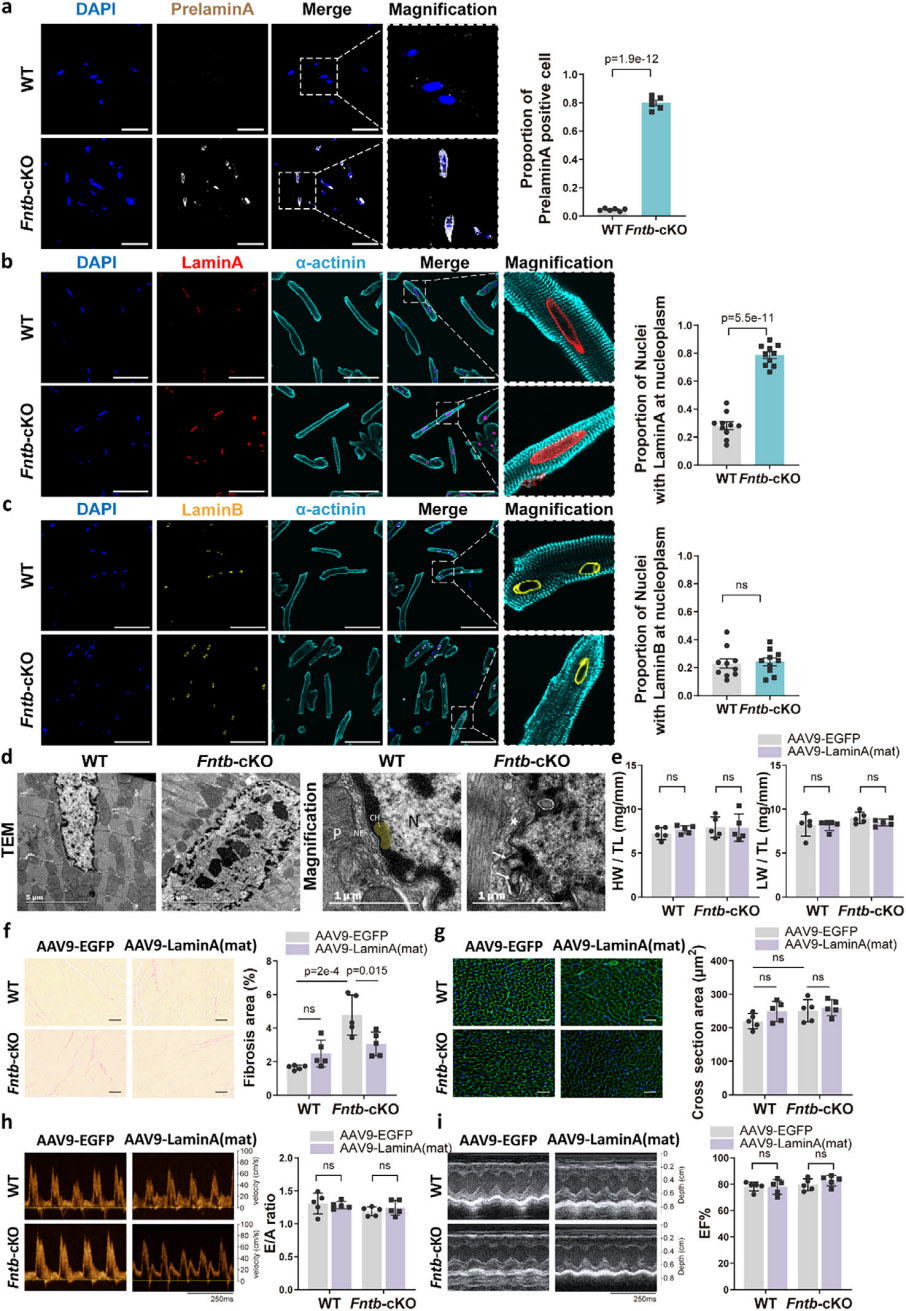
Fntb Knockout Disrupts LaminA Maturation and Nuclear Membrane Integrity in Cardiomyocytes. (a) Immunofluorescence staining with anti‐PrelaminA antibody shows accumulation of PrelaminA in *Fntb*‐cKO AMCMs, scale bar: 50 µm; (b) Immunofluorescence staining with anti‐pan LaminA antibody (detect both precursor and mature forms) shows altered LaminA subcellular localization in *Fntb*‐cKO AMCMs, scale bar: 50 µm; (c) LaminB1 localization remains unaffected in *Fntb*‐cKO cardiomyocytes, scale bar: 50 µm; (d) Transmission electron microscopy (TEM) images reveal nuclear envelope defects in *Fntb*‐cKO cardiomyocytes. The left panel displays irregular nuclear contours with serrated membranes, while the right panel shows discontinuous nuclear membranes (*) and irregular nuclear envelope protrusions (arrows) at higher magnification. Nuclear structures are pseudocolored for clarity: light green (nuclear envelope), light yellow (heterochromatin). Scale bars: 2 µm (left), 500 nm (right). (e) We employed an adeno‐associated virus serotype 9 (AAV9) vector to express the mature form of LaminA (LaminA(mat)). HW and LW normalized to TL in Fntb‐cKO mice treated with AAV9‐EGFP or AAV9‐LaminA(mat) (*n* = 5/group); (f) Representative PSR staining and quantification of interstitial fibrosis, demonstrating that AAV9‐mediated expression of mature LaminA attenuates collagen deposition in *Fntb*‐cKO hearts (*n* = 5/group), scale bar: 50 µm; (g) Representative WGA staining and quantification of cardiomyocyte cross‐sectional area (*n* = 5/group), scale bar: 50 µm; (h,i) Doppler flow assessment of E/A ratio (h) and M‐mode echocardiographic quantification of EF% (i) demonstrating preserved cardiac function across treatment groups (*n* = 5/group). CH: Heterochromatin; N: Nucleus; NE: Nuclear envelope; P: Cytoplasm. Data expressed as mean ± SEM. All data represent mean ± SEM. Multi‐group comparisons were analyzed using two‐way ANOVA with Tukey‐Kramer post hoc test. Two‐group comparisons were performed with an unpaired two‐tailed Student's *t*‐test.

Finally, we investigated the mechanism by which FNTB deletion leads to DNA damage and cardiomyocyte senescence. LaminA, a nuclear envelope protein, has been implicated in the DDR, and its maturation requires farnesylation. In the absence of farnesylation, LaminA remains in an unprocessed precursor form (prelaminA). Consistent with this, immunofluorescence staining for prelaminA demonstrated aberrant accumulation of this precursor in *Fntb*‐cKO cardiomyocytes (Figure 5a). Staining for total LaminA (detecting both mature LaminA and prelaminA) showed aberrant nucleoplasmic LaminA redistribution in 78% of nuclei vs. WT's typical peripheral localization (Figure 5b). This maturation defect was specific to LaminA, as LaminB1 subcellular distribution remained unaltered (Figure 5c). Transmission electron microscopy revealed pronounced ultrastructural abnormalities in FNTB‐deficient cardiomyocyte nuclei. The nuclear envelopes of FNTB knockout nuclei appeared irregular and scalloped, with focal disruptions of the nuclear membrane continuity accompanied by electron‐dense material herniating into the cytoplasm (Figure 5d; Figure S9). These nuclear envelope defects indicate compromised structural stability and are known to trigger DDR activation.

To determine whether defective LaminA maturation is the primary driver of the *Fntb*‐cKO phenotype, we utilized Adeno‐associated virus serotype 9 (AAV9) to overexpress a mature form of LaminA (truncated at amino acid 647), which bypasses the requirement for farnesylation‐dependent processing. Remarkably, the restoration of mature LaminA ameliorated the pronounced interstitial fibrosis characteristic of *Fntb*‐cKO hearts (Figure 5f). Intriguingly, despite the marked attenuation of fibrosis, cardiac hypertrophy as well as global systolic and diastolic functions remained relatively stable during the rescue timeframe (Figure 5e,g–i).

We next examined whether FNTB deletion altered cholesterol metabolism pathway metabolites or enzymes. Counterintuitively, FNTB ablation did not induce FPP accumulation despite the blockade of its consumption in farnesylation. High‐performance liquid chromatography‐mass spectrometry (HPLC‐MS) quantification showed 43% lower cardiac FPP in cKO mice (Figure S10a). This decrease in FPP, however, failed to propagate downstream, as the levels of geranylgeranyl pyrophosphate (GGPP) and cholesterol remained stable (Figure S10a,b). Consistently, the geranylgeranylation status of hallmark substrate proteins, such as RhoA, remained unperturbed by the reduction in FPP levels (Figure S10c). These findings suggest that FNTB deletion is unlikely to drive cardiomyocyte senescence through diminished FPP levels. On the other hand, we observed a significant decrease in the enzymatic activity of HMGCR in *Fntb*‐cKO hearts (Figure S10e), suggesting the reduction in the FPP pool following FNTB loss is likely driven by the impaired activity of HMGCR.

In summary, cardiomyocyte‐specific FNTB deletion impairs LaminA maturation and destabilizes the nuclear envelope, which is likely the primary driver of the DDR observed in FNTB‐deficient hearts.

### 2.5 Lipid Overload Suppresses FNTB Through Srebf2‐Dependent Transcriptional Regulation

**FIGURE 6:**
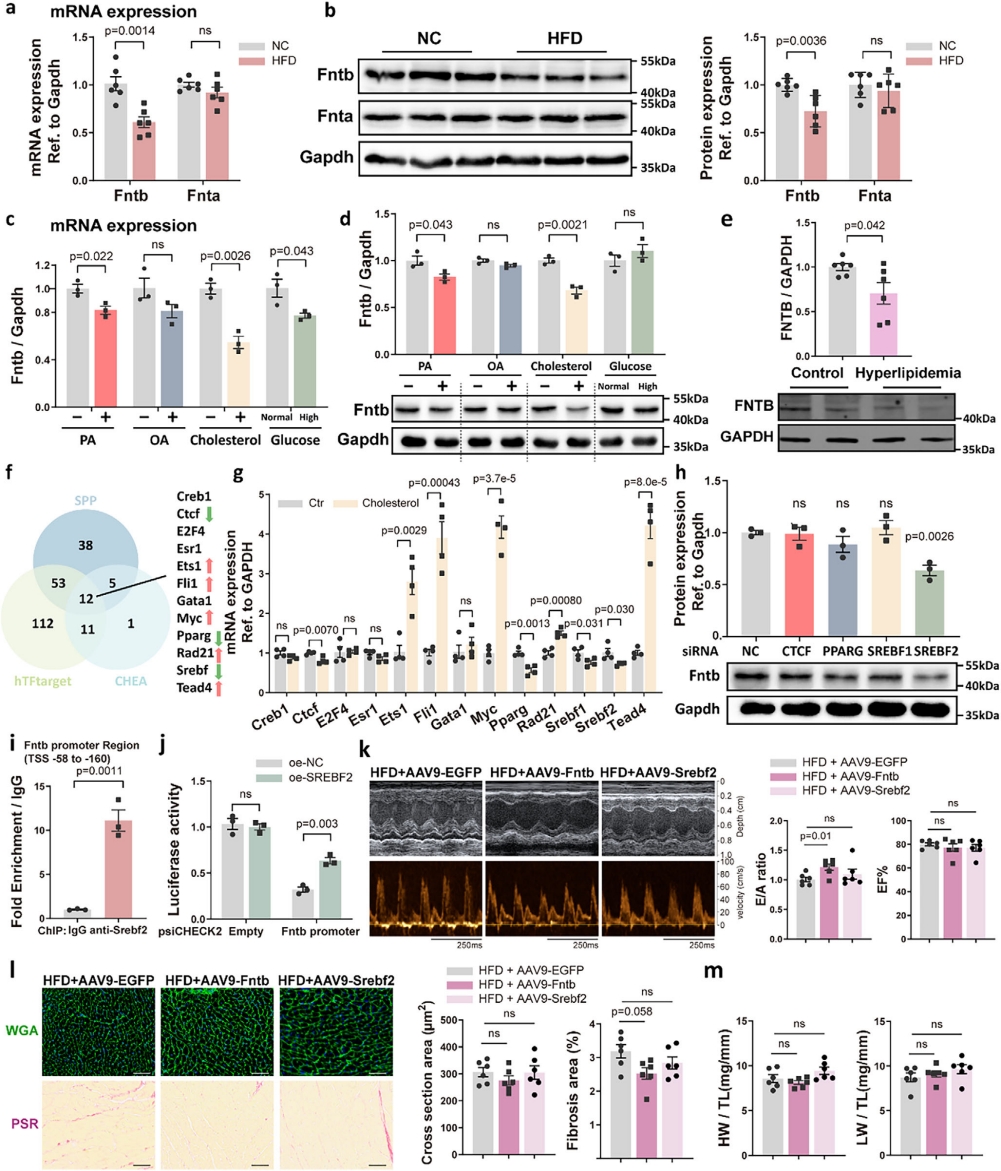
Srebf2 Mediates Cardiomyocyte Fntb Downregulation Under Lipid Overload Conditions. (a) Mice were fed a high‐fat diet (HFD) or normal chow (NC) for 20 weeks. RT‐qPCR analysis of *Fnta* and *Fntb* mRNA in cardiac tissues from HFD‐ or NC‐fed mice (*n* = 6/group); (b) Western blot analysis of Fnta and Fntb protein expression in cardiac tissues from HFD‐ or NC‐fed mice (*n* = 6/group);(c) AMCMs were treated with 500 µm palmitic acid (PA), 500 µm oleic acid (OA), 100 µm cholesterol, or 25 mm glucose. *Fntb* mRNA levels were measured by RT‐qPCR (*n* = 3/group); (d) Western blot analysis of Fntb protein expression in lipid‐stimulated AMCMs (*n* = 3/group); (e) FNTB protein levels were assessed in left ventricular papillary muscle samples from hyperlipidemic and normolipidemic patients (*n* = 6/group);(f) Transcription factors predicted to bind the *Fntb* promoter were identified through three databases (CHEA, hTFtarget, SPP). The left panel shows a Venn diagram of overlapping candidates, while the right panel lists 12 consensus factors. Arrows indicate candidates validated in subsequent experiments; (g) mRNA levels of transcription factor candidates were analyzed in cholesterol‐treated AMCMs (*n* = 4/group); (h) Fntb protein expression was evaluated in AMCMs after siRNA‐mediated knockdown of *Ctcf*, *Pparg*, *Srebf1*, or *Srebf2* (*n* = 3/group); (i) Chromatin immunoprecipitation (ChIP) assays demonstrated Srebf2 binding enrichment at the *Fntb* promoter compared to IgG controls (*n* = 3/group). (j) Dual‐luciferase reporter assay in 293T cells showing the effect of SREBF2 overexpression (oe‐SREBF2) on the transcriptional activity of the *Fntb* promoter (*n* = 3/group); (k) Representative M‐mode and Doppler echocardiographic images (left) with quantification of E/A ratio and EF% (right) in HFD‐fed mice treated with AAV9‐EGFP, AAV9‐Fntb, or AAV9‐Srebf2 (*n* = 6/group); (l) Representative WGA (top) and PSR (bottom) staining with quantification of cardiomyocyte cross‐sectional area and interstitial fibrosis area (*n* = 6/group). Scale bars: 50 µm; (m) HW and LW normalized to TL across the respective experimental groups (*n* = 6/group). All data represent mean ± SEM. Multi‐group comparisons were analyzed using two‐way ANOVA with Tukey‐Kramer post hoc test. Two‐group comparisons were performed with an unpaired two‐tailed Student's *t*‐test.

Given the established link between farnesylation and cholesterol metabolism, we investigated FNTB regulation under lipid overload conditions. In high‐fat diet (HFD)‐fed mice (60% fat, 20 weeks), we observed a distinct cardiac phenotype characterized by interstitial fibrosis, diastolic dysfunction, persistent DNA damage, and induction of senescence markers (Figure S11a–h). In these mice, cardiac Fntb mRNA and protein levels were significantly reduced compared to chow‐fed controls, while Fnta expression remained unaltered (Figure 6a,b). HPLC‐MS analysis showed no significant alterations in cardiac FPP levels following lipid overload, excluding substrate‐mediated feedback mechanisms (Figure S12a). This suppression was recapitulated in cholesterol‐treated primary AMCMs, with palmitate acid (PA) showing partial inhibition (Figure 6c,d; Figure S12b,c). Importantly, left ventricular papillary muscle samples from hyperlipidemic patients showed reduced FNTB protein levels compared to normolipidemic controls (Figure 6e). g To further extend these observations to a broader clinical context, we analyzed publicly available cardiac RNA‐seq data [15] from patients with heart failure with preserved ejection fraction (HFpEF). We found that patients with lower *FNTB* expression exhibited a trend toward increased cardiac fibrosis compared to those in the *FNTB*‐high group (*p* = 0.113; Figure S13a). Moreover, Gene set enrichment analysis (GSEA) demonstrated that the senescence gene set was significantly enriched in the *FNTB*‐low group (Figure S13b).

We next explored the transcriptional mechanism underlying lipid‐induced FNTB suppression. Integrated bioinformatic screening across three independent databases during initial *Fntb* promoter analysis revealed 12 candidate transcription factors, with three (Myc, Srebf, and Ppar) being recognized regulators of lipid metabolism (Figure 6f). Experimental validation in cholesterol‐stimulated cardiomyocytes revealed coordinated downregulation of *Ctcf*, *Pparg*, *Srebf1*, *Srebf2*, and *Fntb* transcripts, whereas other candidates exhibited discordant expression patterns (Figure 6g). Functional interrogation via siRNA knockdown demonstrated specificity—silencing *Srebf2* reduced Fntb protein by 37%, while targeting *Ctcf*, *Pparg*, or *Srebf1* showed no effect (Figure 6h). Direct transcriptional control was confirmed by ChIP‐qPCR showing basal Srebf2 binding at the *Fntb* promoter (Figure 6i). Furthermore, dual‐luciferase reporter assays demonstrated that Srebf2 increases the transcriptional activity of the *Fntb* promoter in 293T cells (Figure 6j).

Finally, we evaluated whether restoring the Srebf2‐Fntb axis could mitigate lipid overload‐induced cardiac remodeling using an AAV9‐mediated overexpression strategy. Notably, AAV9‐Fntb delivery significantly improved diastolic function in HFD‐fed mice, as evidenced by a normalized E/A ratio (Figure 6k), and showed a clear trend toward attenuated interstitial fibrosis (Figure 6l). In contrast, Srebf2 overexpression failed to rescue these pathological parameters, potentially due to its complex downstream metabolic effects or additional lipid‐induced inhibitory signals (Figure 6k,l). Consistent with our previous observations, neither Fntb nor Srebf2 intervention significantly influenced cardiomyocyte hypertrophy or systolic function (Figure 6l,m). These findings identify the suppression of FNTB as a critical driver of diastolic performance and fibrosis under metabolic stress.

Together, these results demonstrate that lipid overload reduces cardiomyocyte FNTB expression through SREBF2‐mediated transcriptional suppression, subsequently decreasing farnesylation.

## 3 Discussion

Cardiomyocyte senescence plays a pivotal role in cardiac remodeling, yet the regulatory mechanisms remain poorly understood. Our findings identify FNTB as a critical orchestrator of cardiomyocyte senescence. Exposure to high‐fat conditions reduces farnesylation in cardiomyocytes, impairing laminA maturation and inducing DNA damage responses. This, in turn, triggers premature cardiomyocyte senescence characterized by secretion of profibrotic factors such as Tgf‐β2 and Gdf15, promoting fibroblast activation and cardiac fibrosis.

Recent studies have indeed highlighted the contribution of lipid overload to cardiomyocyte senescence. Our findings indicate that excess lipid accumulation impairs FNTB expression in cardiomyocytes. This defect hinders laminA processing, thereby perpetuating fibrotic remodeling through chronic SASP activation. Importantly, clinical observations of moderate FNTB reduction in hyperlipidemic human hearts suggest these pathogenic effects may manifest more gradually than in our murine knockout model. Notably, a vicious cycle may emerge in metabolic heart disease: Lipid accumulation suppresses FNTB to induce senescence, while senescent cells exhibit impaired cholesterol handling and Srebf2 suppression [16], further exacerbating lipid dysregulation.

Regarding the role of farnesylation in cardiac remodeling, we observed that FNTB‐deficient hearts maintained systolic function despite progressive fibrosis, recapitulating clinical features of lipotoxic cardiomyopathy. However, pressure overload unmasked latent cardiac dysfunction, with knockout mice exhibiting accelerated heart failure. This phenotypic divergence may arise because early‐stage senescent cardiomyocytes—present in limited numbers—predominantly influence neighboring fibroblasts through SASP rather than directly impairing contractile function. Nevertheless, this early senescent environment ultimately compromises cardiac resilience to superimposed stress. On the other hand, our previous research demonstrated that adenovirus‐mediated overexpression of *Fntb* in neonatal rat cardiomyocytes induced hypertrophy, apoptosis, and pathological autophagy, largely due to hyperactivation of Ras proteins [17]. These earlier findings align with our current observations, where FNTB ablation reduces Ras activity, enhances apoptosis resistance, and diminishes autophagic flux. The absence of prominent hypertrophy in *Fntb*‐knockout cardiomyocytes may stem from diminished Ras signaling, given its established role in driving hypertrophic growth [18]. While both excessive and insufficient farnesylation disrupt cardiac homeostasis, our data suggest that restoring physiological farnesylation or activating Srebf2 may represent a therapeutic strategy for obesity‐associated cardiac remodeling, though this requires validation in metabolic models.

While our data strongly implicates SASP as the primary driver of the observed fibrotic remodeling, we rigorously evaluated the potential contributions of alternative pathological pathways. Within the established paradigms of myocardial pathology, fibrosis typically manifests in two distinct forms: reparative fibrosis, which compensates for cardiomyocyte attrition, and reactive fibrosis, which is often orchestrated by inflammatory cell infiltration, hemodynamic instability, or aberrant mechanical signaling. However, several lines of evidence in our model argue against these conventional mechanisms. Specifically, the marked resistance to apoptosis, the absence of overt inflammatory cell recruitment, and the emergence of fibrotic lesions prior to any detectable hemodynamic dysfunction collectively suggest that SASP‐independent drivers are unlikely to be the predominant initiators of the remodeling process. Complementary insights from WGCNA revealed that genes within modules 3 and 4 were significantly enriched in pathways related to autophagy impairment and mitochondrial transcriptional arrest, respectively. These cellular perturbations can be interpreted either as intrinsic hallmarks of the senescence program or as autonomous profibrotic processes. Consequently, while these factors cannot be entirely discounted, their potential roles as independent drivers of cardiac remodeling warrant further granular investigation.

The temporal progression of senescence markers in our model warrants particular attention. Early DNA damage responses and SASP emergence (within 2 weeks) preceded classical senescence markers like SA‐β‐gal accumulation (24 weeks), supporting the concept that senescence comprises distinct phases with different biomarker trajectories. And it is important to note that the emergence of SASP does not require concurrent activation of all senescence markers, as markers like cell cycle arrest and lysosomal activity changes do not directly cause SASP [19, 20]. Instead, DNA damage responses are primary drivers of SASP in senescent cells [21], potentially explaining our early observations. Nevertheless, we explicitly acknowledge the inherent limitations of relying on classical markers such as SA‐β‐gal and Cdkn2a. These indicators lack absolute specificity for the senescent state and are essentially surrogates for physiological changes that are susceptible to modulation by cellular metabolic status, cell type, and functional alterations (e.g., lysosomal expansion or autophagy). This biological ambiguity introduces potential confounding factors that could affect the accuracy of our conclusions regarding the definitive classification of the cardiomyocyte senescence phenotype.

The aberrations in LaminA structure and function and their impact on DNA damage response and cellular senescence have been extensively documented [22, 23, 24]. Electron microscopy in our study revealed nuclear envelope ultrastructural abnormalities, such as blebbing and rupture, consistent with those observed in LaminA mutation‐associated diseases like Emery‐Dreifuss muscular dystrophy, Hutchinson‐Gilford progeria syndrome (HGPS), and dilated cardiomyopathy [25, 26, 27]. These findings support a causal role for LaminA maturation defects following FNTB deletion. Nonetheless, it should be acknowledged that our focus on LaminA as the primary downstream effector was based on a candidate‐driven approach rather than high‐throughput screening, such as farnesyl‐proteomics. Consequently, the potential contribution of other isoprenylated proteins to the observed phenotypes cannot be entirely ruled out. Furthermore, while the role of LaminA is well‐characterized in rare genetic syndromes, its regulatory significance in non‐heritable, age‐related diseases remains largely underexplored. Elevated PreLaminA in aging populations has been linked to DNA damage and vascular calcification [28], while gut microbiota dysbiosis‐induced Nod2 activation leading to LaminA degradation has been associated with hepatic DNA damage and carcinogenesis [29]. Our findings highlight the involvement of the FNTB‐LaminA axis in the context of cardiomyocyte senescence. Importantly, farnesyltransferase inhibitors like lonafarnib, currently approved for treating HGPS [30], highlight the complex roles of farnesylation. Blocking farnesylation reduces the toxicity of progerin by preventing its nuclear membrane localization [31]. However, non‐farnesylated LaminA retains residual toxicity [32, 33], which may explain why farnesyltransferase inhibitors alleviate HGPS symptoms but fail to effect a complete cure [34].

While our study underscores the structural compromise of the nuclear envelope as a central driver of genomic instability, it is imperative to critically evaluate alternative mechanisms, including Reactive oxygen species (ROS) accumulation and metabolic toxicity. ROS are classical endogenous mutagens capable of inflicting direct oxidative DNA damage [35]. However, the interplay between farnesylation and ROS is complex; while the activation of Ras has been causally linked to ROS generation [36], FNTB deficiency canonically inhibits Ras farnesylation and membrane anchoring. This loss of function would theoretically suppress rather than enhance Ras signaling, arguing against Ras‐mediated ROS as a primary instigator in our model. Similarly, while cytotoxic lipid peroxidation adducts derived from excessive fatty acid oxidation are known to compromise genomic integrity [37], our metabolomic profiling revealed no significant aberrations in lipid metabolism following FNTB deletion. Therefore, metabolic lipotoxicity appears unlikely to be the initial trigger. Nevertheless, we cannot entirely exclude the involvement of other factors, such as defects in DNA repair machinery recruitment or transcription‐replication conflicts, which may operate in concert with nuclear envelope instability to fuel the DNA damage response.

Despite these insights, several limitations of the present study should be acknowledged. First, while we propose that senescent cardiomyocytes and their SASP drive fibrotic phenotypes, we did not directly demonstrate causality by selectively eliminating senescent cells in vivo. Second, longer‐term studies on cardiomyocyte‐specific FNTB deficiency under chronic stress are necessary to fully understand its aging trajectory. Third, regarding the translational relevance, we observed reduced FNTB expression in heart tissues from hyperlipidemic patients; however, we acknowledge that these findings are correlative. Caution must be exercised in extrapolating our murine mechanistic findings directly to human pathology.

## 4 Methods

### 4.1 Mouse Models

All animal procedures were conducted in accordance with the national guidelines for experimental animal welfare and were approved by the Committee for Experimental Animal Science of Zhejiang University (Approval No: ZJU20200102). Mice were housed in a specific pathogen‐free facility at the Zhejiang University Animal Experiment Center under controlled environmental conditions (22°C–24°C, 40%–60% humidity) with a standardized 12‐h light/dark cycle.

*Fntb*‐floxed mice on a C57BL/6 background and Myh6‐MerCreMer transgenic mice were obtained from GemPharmatech Co., Ltd. To generate cardiomyocyte‐specific *Fntb* knockout mice, *Fntb* fl/fl mice were crossed with Myh6‐MerCreMer (+) transgenic mice. Male Myh6‐MerCreMer (+) *Fntb* ^fl/fl^ mice at 6 weeks of age received intraperitoneal tamoxifen injections (30 mg/kg body weight) for five consecutive days to induce Cre‐mediated recombination and FNTB deletion. Myh6‐MerCreMer (‐) *Fntb* ^fl/fl^ littermates injected with equivalent tamoxifen doses served as controls. Genotypic analysis confirmed that *Fntb* knockout offspring followed Mendelian inheritance ratios without gross developmental abnormalities.

### 4.2 Construction of Lipid Overload Mouse Model

8‐weeks‐old male wild‐type C57BL/6 mice were randomly assigned to receive either a HFD (60 kcal% fat, 20 kcal% protein, 20 kcal% carbohydrates; D12492, Research Diets Inc.) or a standard control diet (10 kcal% fat, 20 kcal% protein, 70 kcal% carbohydrates; D12450B, Research Diets Inc.) for 20 weeks. A subset of mice pre‐fed with these respective dietary regimens was obtained from Cavance Co., Ltd.

### 4.3 AAV9 Vector Construction, Virus Packaging, and Tail Vein Injection Intervention

To achieve cardiomyocyte‐specific overexpression, this study utilized an AAV9 system, with expression driven by the cardiomyocyte‐specific cTNT or TNT promoter. The target genes included mouse *Fntb*, *Srebf2*, and a truncated mutant of *Lmna*. For the *Lmna* vector, a truncated mutant mimicking the mature form of LaminA was constructed by introducing a stop codon after Tyrosine (Y) at position 861 within the C‐terminal RSY motif of the mature sequence.

AAV packaging was performed by Hanbio Biotechnology Co., Ltd. A triple‐plasmid co‐transfection system was employed, where a shuttle plasmid carrying the target gene was co‐transfected into AAV‐293 cells along with the pAAV‐RC plasmid (encoding rep and cap proteins) and the pHelper plasmid. Cells were harvested 72 h post‐transfection, and virus particles were released via three freeze–thaw cycles. After treatment with Benzonase to remove residual DNA, the viral particles were purified through column chromatography and concentrated via ultrafiltration to obtain high‐titer stocks. All viral batches were verified for the absence of bacterial, fungal, and mycoplasma contamination using microscopy and PCR assays. Viral genomic titers were determined by qPCR and ranged from 1.5*10^12^–2.3*10^12^ vg/mL.

For in vivo gene intervention, viral particles were administered via tail vein injection. An AAV9 vector carrying enhanced green fluorescent protein (EGFP) served as the control. The injection dose for both target and control viruses was maintained at approximately 1*10^11^–1*10^12^ vg per mouse. In the rescue experiments, the AAV9 virus carrying the *Lmna*(mat) gene was injected into *Fntb*‐cKO mice on the first day of tamoxifen induction; mice were sacrificed for analysis 4 weeks post‐injection. Wild‐type C57BL/6 mice were fed an HFD for 12 weeks, followed by tail vein injection of AAV9‐*Fntb*, AAV9‐*Srebf2*, or the control virus. The mice continued receiving the HFD for an additional 8 weeks before being sacrificed for sample collection.

### 4.4 TAC

8‐weeks‐old male *Fntb*‐cKO and WT mice were randomized to TAC or sham surgery. Under anesthesia with 1% pentobarbital sodium (100 mg/kg, i.p.), a midline sternotomy exposed the aortic arch. For TAC, a 27‐gauge needle was placed adjacent to the aortic arch and ligated with two 6‐0 polypropylene sutures, generating a 0.4 mm constriction upon needle removal. Sham mice underwent identical dissection without ligation. Postoperative care included analgesia and 4‐weeks monitoring prior to terminal echocardiography and tissue harvest.

### 4.5 DDR Inhibition Treatment

The DDR inhibitor, KU55933, was dissolved in saline containing 10% DMSO as a vehicle. Beginning 2 days post‐tamoxifen induction, WT and *Fntb*‐cKO mice received intraperitoneal injections of KU55933 (5 mg/kg) every other day for 12 weeks. Control cohorts were administered equivalent volumes of vehicle (10% DMSO in saline) on the same schedule. Terminal tissue collection followed treatment completion.

### 4.6 Primary Adult Mouse Cardiomyocyte (AMCM) Isolation and Cultivation

Primary AMCMs isolation was performed based on the protocol established by Ackers‐Johnson et al. [38] with two critical modifications: retrograde aortic perfusion optimization and enzymatic digestion adjustment. Briefly, excised hearts were secured at the aortic root using a blunt needle and 6‐0 silk sutures, followed by sequential coronary perfusion: (1) ice‐cold EDTA buffer (pH 7.4) to evacuate blood components, (2) complete drainage of EDTA buffer, and (3) continuous recirculation of pre‐warmed enzymatic digestion buffer (37°C; 1 mg/mL collagenase type II, 0.05 mg/mL protease XIV in Ca^2^^+^‐free medium) for 20–25 min, with digestion duration dynamically adjusted based on tissue softening and fibrosis severity. The enzymatically treated hearts were minced into 1 mm^3^ fragments, gently triturated using Pasteur pipettes for 5 min, and quenched with stop buffer containing 10% fetal bovine serum. The cell suspension was filtered through a 100 µm nylon mesh to remove undigested tissue, followed by two rounds of gravity sedimentation (20 min each) to enrich cardiomyocytes, which were then subjected to stepwise Ca^2^^+^ reintroduction (0.2, 0.5, and 1.2 mm) to restore physiological calcium tolerance before plating onto laminin‐coated dishes. For cardiac fibroblast isolation, the supernatant from the first sedimentation was further clarified by an additional 10‐min sedimentation to pellet residual cardiomyocytes, after which the secondary supernatant was centrifuged (300 × g, 5 min) and the pellet resuspended in fibroblast growth medium. Both cell types were maintained under standard culture conditions.

### 4.7 Lipid Overload and High‐Glucose Stimulation in AMCM

For lipid overload studies, PA, oleic acid (OA), and cholesterol were individually administered to cardiomyocytes. PA and OA stock solutions (5 mm complexed with 10% fatty acid‐free BSA) were diluted in fatty acid‐free culture medium to final concentrations of 500 µm (1% BSA) [39], while water‐soluble cholesterol powder (40 mg cholesterol per 1 g powder) was dissolved in fatty acid‐free medium to achieve 100 µm cholesterol [40]. Cells were pre‐incubated in fatty acid‐free medium for 1 h before 48‐h exposure to lipid‐supplemented media. Control groups for PA/OA treatments received 1% BSA‐containing medium, whereas cholesterol controls utilized unmodified fatty acid‐free medium.

For high‐glucose stimulation, glucose powder was added to standard M199 medium (basal glucose: 5 mm) to achieve a final concentration of 25 mm. Cells underwent 1‐h acclimation in standard medium (5 mm glucose) prior to 48‐h treatment with high‐glucose medium, while controls remained in unmodified medium. All experiments were conducted under serum‐free conditions, with endpoint analyses performed post‐stimulation.

### 4.8 siRNA Treatment

Gene‐specific siRNAs targeting *Ctcf*, *Ppparg*, *Srebf1*, *Srebf2*, *Tgfb2*, *Gdf15*, *Hsp70*, and *Bcl2* along with a non‐targeting negative control siRNA (NC‐siRNA), were synthesized by GenePharma Co., Ltd. (sequence details in Table S1). Primary AMCMs and NRCMs were isolated and seeded in 6‐well plates. Cells were transfected with 50 nm siRNA using Lipofectamine RNAiMAX (Thermo Fisher Scientific), strictly adhering to the manufacturer's reverse transfection protocol. Transfection complexes were prepared at a 1:1 (v/v) ratio of Lipofectamine to siRNA in serum‐free Opti‐MEM medium, followed by 20‐min incubation at room temperature. Gene silencing efficiency was assessed 24–48 h post‐transfection via RT‐qPCR and immunoblotting.

### 4.9 Primary Neonatal Rat Cardiomyocytes (NRCMs) Isolation and Adv‐shRNA Transfection

NRCMs were isolated from postnatal day 0 (P0) rats using the Neonatal Rat Cardiomyocyte Isolation Kit (Miltenyi Biotec, #130‐098‐373). The heart tissue underwent three cycles of enzymatic digestion at 37°C (15 min per cycle), followed by filtration through a 70 µm cell strainer and centrifugation. To ensure cardiomyocyte purity, fibroblasts were removed via differential plating for 20 min, and 5‐bromo‐2'‐deoxyuridine (BrdU, 1:1000) was supplemented in the culture medium. 20 h after seeding, cells were transfected with recombinant adenoviruses (Adv‐shFntb or Adv‐GFP) generated using the pAdMax system. The viruses were incubated with the cells in serum‐free high‐glucose DMEM for 2 h, after which the medium was replaced with complete medium containing 10% Fetal Bovine Serum (FBS) for further cultivation.

### 4.10 Genotyping Procedure

Genomic DNA was isolated from mouse tail snips and toe tip biopsies using standard phenol‐chloroform extraction, followed by PCR amplification of the target loci with forward primer 5’‐AAAGGCACTCACCAATAAACT‐3’and reverse primer 5’‐TATGGCAGTCAGAAGTCAGCT‐3’. Thermal cycling conditions comprised an initial denaturation at 95°C for 5 min, 35 cycles of denaturation (95°C, 30 sec), annealing (58°C, 30 sec), and extension (72°C, 30 sec), with a final extension at 72°C for 5 min. Amplified products were electrophoresed on 1% agarose gels and visualized using a Tanon 2500 Gel Imaging System. Genotyping was determined by distinct band sizes: a 455 bp fragment indicated the wild‐type allele, while a 592 bp fragment corresponded to the *Fntb* flox allele.

### 4.11 Mouse Echocardiography

Following anesthesia with 1% pentobarbital sodium, mice were positioned supine on a temperature‐controlled platform, and thoracic fur was removed. Transthoracic echocardiography was performed using a Vevo 2100 High‐Resolution Imaging System (Visual Sonics, Toronto, Canada) equipped with a 40 mHz MS‐550D linear transducer. 2D M‐mode imaging in the parasternal short‐axis view at the papillary muscle level captured ≥5 consecutive cardiac cycles. Key parameters including left ventricular posterior wall thickness, interventricular septal thickness, left ventricular internal diameter in diastole, and systole were measured offline. Left ventricular FS and EF were calculated using the above primary measurements and accompanying software. All image acquisition and analyses were conducted by board‐certified sonographers blinded to experimental group assignments.

To evaluate left ventricular diastolic function, pulsed‐wave (PW) Doppler imaging was performed in the apical four‐chamber view. The Doppler sample volume was precisely positioned at the tips of the mitral valve leaflets to record the mitral inflow velocity profile. From the resulting Doppler spectra, the peak early diastolic filling velocity (E) and the peak late (atrial) diastolic filling velocity (A) were measured over ≥5 consecutive cardiac cycles. The E/A ratio was subsequently calculated to quantify diastolic performance. Similar to the systolic analysis, all PW Doppler measurements were conducted offline using the Vevo 2100 software by sonographers blinded to the experimental assignments.

### 4.12 RT‐qPCR

Total RNA was isolated using TRIzol reagent (Invitrogen) following the manufacturer's protocol. First‐strand cDNA synthesis was performed with 1 µg total RNA using the PrimeScript RT Reagent Kit (Takara Bio, #RR037A). Quantitative PCR reactions contained cDNA template, gene‐specific primers (Table S2), and TB Green Premix Ex Taq II (Takara Bio, #RR420A). Amplification was carried out on a LightCycler 480 II system (Roche) under the following conditions: 95°C for 5 min (initial denaturation); 40 cycles of 95°C for 15 sec, 58°C for 20 sec (annealing), and 72°C for 30 sec (extension); followed by melt curve analysis (65°C–95°C, 0.5°C increments). Reactions were performed in technical triplicate. Relative mRNA expression was normalized to Gapdh using the 2^−ΔΔCt^ method and expressed as fold change vs. control groups.

### 4.13 Western Blot Analysis

Protein lysates were prepared using RIPA or Lysis buffer supplemented with protease/phosphatase inhibitor cocktails (Roche). Protein concentrations were quantified via BCA assay, and equal amounts (10–40 µg) were resolved on 7%–12% SDS‐polyacrylamide gels. Separated proteins were transferred to PVDF membranes (chemiluminescence detection) or nitrocellulose membranes (fluorescence detection) using a Bio‐Rad Trans‐Blot Turbo system. Membranes were blocked with 5% non‐fat milk in TBST (Tris‐buffered saline with 0.1% Tween‐20) for 1 h at room temperature, then incubated overnight at 4°C with primary antibodies (Table S3). For chemiluminescent detection, membranes were probed with HRP‐conjugated secondary antibodies and developed using ECL Chemiluminescent Substrate on a ChemiDoc MP Imaging System (Bio‐Rad). Fluorescence‐based detection utilized IRDye‐conjugated secondary antibodies with imaging on an Odyssey CLx system (LI‐COR). Band intensities were normalized to β‐Tubulin or Gadph and quantified using ImageJ (v1.51j) or Image Studio Lite (v5.2).

### 4.14 Histological Analysis

Cardiac tissues were excised and immersion‐fixed in 4% paraformaldehyde (PFA) for a minimum of 24 h, followed by sequential dehydration in graded ethanol solutions, paraffin embedding, and sectioning at 5 µm thickness. For histological staining, sections were deparaffinized in xylene and rehydrated through a graded ethanol series. WGA staining was performed by incubating sections with Alexa Fluor 488‐conjugated WGA for 30 min at room temperature in darkness. PSR staining was conducted by treating sections with 0.1% Sirius Red in saturated picric acid, followed by acidified water rinses. TUNEL staining was employed to detect apoptotic cells; briefly, rehydrated sections were permeabilized with Proteinase K, followed by incubation with the TUNEL reaction mixture at 37°C for 1 h and DAPI counterstaining. All slides were imaged using an Olympus BX63 epifluorescence microscope. Cardiomyocyte cross‐sectional area and collagen deposition were quantified using Adobe Photoshop (v2021), blinded to experimental groups.

### 4.15 Immunofluorescent Staining

Cardiomyocytes and cardiac fibroblasts isolated from adult mice were fixed under optimized conditions: 4% PFA for 15 min at room temperature for general markers, or ice‐cold acetone (−20°C) for 10 min specifically for prelaminA detection. Following three PBS washes, cells were permeabilized with 0.5% Triton X‐100 for 10–20 min and blocked with 10% normal sheep serum in PBS for 1 h. Primary antibodies (detailed in Table S3) were applied in blocking buffer at 4°C overnight. After three PBST (0.1% Tween 20) washes, species‐matched Alexa Fluor‐conjugated secondary antibodies were incubated for 1 h at room temperature protected from light. Nuclei were counterstained with DAPI for 5 min. Images were acquired using a Zeiss LSM 880 confocal microscope. Quantitative analysis of fluorescence intensity and subcellular localization was performed using Adobe Photoshop (v2021) and ImageJ (v1.51j) by investigators blinded to experimental conditions.

Paraffin‐embedded heart sections (5 µm) were deparaffinized, rehydrated, and subjected to heat‐induced antigen retrieval in 10 mm citrate buffer (pH 6.0). After blocking with 10% normal sheep serum, sections were incubated overnight at 4°C with primary antibodies against F4/80 and CD45. Species‐matched Alexa Fluor‐conjugated secondary antibodies were used for detection, followed by DAPI counterstaining. Confocal images (Zeiss LSM 880) were quantified using ImageJ by investigators blinded to experimental group assignments.

### 4.16 Transcriptome Sequencing

Cardiomyocytes were isolated from three WT and three *Fntb*‐cKO mice at 14 days post‐tamoxifen induction. Total RNA was extracted with TRIzol Reagent and subjected to quality control. Strand‐specific mRNA sequencing libraries were prepared using the NEBNext Ultra II Directional RNA Library Prep Kit (Illumina). RNA sequencing services were performed by Novogene Corporation (Beijing, China) on an Illumina NovaSeq platform, generating 150 bp paired‐end reads. Raw reads were processed through Novogene's in‐house pipeline: adapter trimming, quality filtering, and alignment to the GRCm38/mm10 reference genome via HISAT2 (v2.0.5) with default parameters. Gene‐level quantification was performed using featureCounts with strand‐specific counting, and normalized expression values were calculated accounting for gene length and sequencing depth.

### 4.17 Bioinformatics Analysis

Differential gene expression analysis was performed using DESeq2 (v1.30.0) in R (v4.0.3), with significance defined as Benjamini‐Hochberg adjusted *p* < 0.05.

WGCNA was implemented using the WGCNA R package (v1.72). As suggested by the authors, the top 25% most variable genes were selected for network construction. A scale‐free topology was achieved by applying a soft threshold power (β = 29) determined through scale‐free topology fit analysis (R^2^> 0.85). The adjacency matrix was transformed into a topological overlap matrix (TOM) to minimize spurious correlations, followed by hierarchical clustering with dynamic hybrid tree‐cutting (minModuleSize = 30, deepSplit = 2, mergeCutHeight = 0.4). Gene modules were assigned unique color labels, and module‐trait associations were quantified via Pearson correlation between module eigengenes and genotype status.

Pathway enrichment analysis was conducted using the PANTHER classification system (v17.0, http://www.pantherdb.org) with Reactome Pathways. Gene lists from the module of interest were subjected to statistical overrepresentation testing using Fisher's exact test with false discovery rate (FDR) correction. Significantly enriched pathways (FDR< 0.05) were hierarchically organized by semantic similarity and filtered to prioritize mid‐level annotations (clustering tiers 2–4) to avoid overgeneralization, following established practices for functional interpretation [41]. Enrichment results were visualized as bubble plots ranked by fold enrichment.

To assess the clinical relevance of our findings, we analyzed publicly available cardiac RNA‐seq data and clinical metadata from a heart failure cohort described by Hahn et al. [15]. Among the study population, we specifically selected 41 patients with HFpEF for further analysis. 41 Patients were stratified into three groups based on the tertiles of *FNTB* raw counts: Low (bottom 33%), Medium (middle 33%), and High (top 33%). Cardiac fibrosis scores, provided by the original study metadata, were compared across these groups.

GSEA was performed to identify biological signatures associated with *FNTB* expression levels. We utilized the SenMayo gene set, a consensus panel of 125 senescence‐associated genes proposed by Saul et al., [43] to evaluate the cellular senescence status in *FNTB*‐low vs. *FNTB*‐high HFpEF hearts. The analysis was conducted using the R package clusterProfiler (v4.18.4) within the R statistical environment (v4.5.2).

### 4.18 Identification and Validation of Fntb Promoter‐Binding Transcription Factors

To identify transcription factors regulating *Fntb* expression, we integrated computational predictions from three databases: ChIP‐X Enrichment Analysis (ChEA3, http://amp.pharm.mssm.edu/lib/chea.jsp), hTFtarget (http://bioinfo.life.hust.edu.cn/hTFtarget), and the Signaling Pathways Project (SPP, https://www.signaling‐pathways.org). Candidates common to all three platforms were functionally screened via siRNA‐mediated knockdown in AMCM, followed by RT‐qPCR quantification of *Fntb* mRNA levels.

For validated transcription factors, putative binding motifs were predicted using JASPAR (https://jaspar.genereg.net), and primers flanking these motifs were designed for ChIP validation. ChIP assays were performed using the SimpleChIP Enzymatic Chromatin IP Kit (#9005, Cell Signaling Technology). AMCMs were crosslinked with 1% formaldehyde for 10 min at 25°C, quenched with 125 mm glycine, and nuclei isolated using ice‐cold PBS supplemented with protease inhibitors. Chromatin was enzymatically digested with Micrococcal Nuclease (37°C, 20 min) to generate 200–500 bp fragments, followed by nuclear lysis via ultrasonication. Cleared chromatin lysates were immunoprecipitated overnight at 4°C with 2 µg anti‐SREBF2 antibody or rabbit IgG control, coupled to Protein G Magnetic Beads. After sequential washes with low‐ and high‐salt buffers, bound chromatin was eluted in ChIP elution buffer, reverse‐crosslinked with 200 mm NaCl and proteinase K, and purified using silica‐membrane columns. Enrichment of target DNA fragments was quantified by qPCR using primers flanking predicted binding motifs.

### 4.19 Isoprenoid Quantification by HPLC‐MS

Cardiac tissue samples (40–60 mg) were rinsed with 100 mM NH_4_HCO_3_ and homogenized in a 1:1(v/v) mixture of NH_4_HCO_3_ and isopropanol. After centrifugation, the supernatant was mixed with acetonitrile, dried under nitrogen gas at 40°C, and reconstituted in 50% methanol. Chromatographic separation was performed on an Agilent XDB C18 column using an Agilent 6460 Triple Quadrupole LC/MS system equipped with electrospray ionization. FPP, geranyl pyrophosphate (GPP), and GGPP levels were quantified via HPLC‐MS. Calibration curves were constructed using authentic standards (Sigma–Aldrich). Tissue concentrations were normalized to wet weight. Detailed methodological protocols are described in our prior publication [42].

### 4.20 SA‐β‐Gal Staining

SA‐β‐Gal activity was detected using the Cellular Senescence Detection Kit (Dojindo Molecular Technologies, #SG03). Freshly isolated AMCMs were plated on laminin‐coated dishes and fixed with 4% formaldehyde for 3 min at room temperature. Cells were incubated with SPiDER‐βGal working solution (1:1,000 dilution in McIlvaine buffer, pH 6.0) for 30 min at 37°C under ambient atmosphere. Fluorescent images were acquired using a Zeiss LSM 880 confocal microscope (excitation/emission: 488/520 nm). Nuclei with SPiDER‐βGal fluorescence signal were classified as SA‐β‐Gal‐positive. Quantification was performed by blinded investigators counting ≥100 cells per condition across three biological replicates, with results expressed as a percentage of positive cells.

### 4.21 Conditioned Media Transfer

Cardiomyocytes isolated from WT and *Fntb*‐cKO mice at 14 days post‐tamoxifen administration were plated on laminin‐coated dishes and allowed to adhere for 4 h in culture medium. After attachment, cells were washed twice with PBS and incubated in serum‐free cardiomyocyte maintenance medium for 24 h at 37°C (5% CO_2_). Conditioned media were collected, centrifuged to remove cellular debris, and aliquoted into two treatment groups: incubated at 100°C for 10 min followed by rapid cooling on ice for 5 min; (2) maintained on ice for 15 min without thermal denaturation.

Primary cardiac fibroblasts were isolated from adult WT mice via enzymatic digestion, expanded for one passage in complete medium, and synchronized by serum gradient depletion. Following 6 h of serum‐free conditioning, fibroblasts were treated with cardiomyocyte‐conditioned media for 24 h. The subsequent analyses performed on these samples included the CCK‐8 assay, immunofluorescence staining, and RNA extraction.

### 4.22 ELISA

Cardiomyocytes isolated from WT and *Fntb*‐cKO mice at 14 days post‐tamoxifen administration were plated on laminin‐coated dishes and cultured in serum‐free cardiomyocyte medium for 24 h under standard conditions (37°C, 5% CO_2_). Then medium was centrifuged to remove cellular debris. Concentrations of secreted GDF15 and TGF‐β2 were quantified using commercial ELISA kits following manufacturer protocols.

### 4.23 Transmission Electron Microscopy (TEM) Analysis

Heart tissue samples were immediately fixed in 2.5% glutaraldehyde solution for 4 h at room temperature, followed by post‐fixation in 1% osmium tetroxide for 1 h and en bloc staining with 2% uranyl acetate for 30 min. Dehydration was performed through an ethanol series (50%, 70%, 90%, and 100%) before embedding in epoxy resin. Ultrathin sections (120 nm) were cut using an ultramicrotome, mounted on copper grids, and double‐stained with 4% uranyl acetate (20 min) and lead citrate (5 min). Ultrastructural examination of the heart tissue was carried out using a transmission electron microscope.

### 4.24 Dual‐Luciferase Reporter Assay

The promoter sequence of the *Fntb* gene was amplified and subcloned into the psiCHECK‐2 dual‐luciferase reporter vector (Promega). The insertion sites were engineered using the restriction enzymes Kpn I and Nhe I. The resulting construct was designated as FNTB promoter‐psiCHECK 2. The final constructed plasmid was verified by sequencing.

For the functional validation, 293T cells were cultured in DMEM medium supplemented with 10% FBS. Cells were plated in 24‐well plates and co‐transfected with 0.8 µg of the reporter plasmid (either the *Fntb* promoter‐psiCHECK‐2 or the empty psiCHECK‐2 control) and an overexpression plasmid (either the *SREBF2* overexpression plasmid, oe‐SREBF2, or the negative control, oe‐NC). Transfection was performed using LipoFiter. After 48 h, cells were lysed, and the luciferase activities were measured using the Dual‐Luciferase Reporter Assay System (Promega, Cat. No. E1910). Relative luciferase activity was calculated as the ratio of Firefly luciferase activity to Renilla luciferase activity, which was used to normalize for differences in cell viability and transfection efficiency.

### 4.25 Detection of RhoA Geranylgeranylation via Click Chemistry

To assess RhoA geranylgeranylation, cell lysates were first pre‐cleared with streptavidin beads (1 mg/mL) at 4°C for 2 h to remove endogenous biotin. The click reaction was then performed for 2 h at room temperature in a 500 µL system containing 1 µg/µL protein, 50 µm biotin‐alkyne probe, 1 mm CuSO_4_, 0.1 mm TBTA, and 4 mm NaVc. Proteins were precipitated using the methanol‐chloroform method at −80°C, washed with cold methanol, and resuspended in 1% SDS. After adjusting the SDS concentration to 0.3% with NP‐40 buffer, geranylgeranylated proteins were enriched using streptavidin magnetic beads overnight at 4°C. The beads were washed sequentially with PBS, 6 m urea, and PBS, followed by elution in 1× loading buffer at 100°C for 10 min. The enriched RhoA was finally detected via immunoblotting.

### 4.26 HMGCR Activity Assay

HMGCR activity in heart tissues was evaluated using a commercial kit (Solarbio, BC0490) according to the manufacturer's protocol. Briefly, tissue supernatants were prepared using the provided extraction buffer. The enzymatic reaction was initiated by mixing the samples with a working solution containing HMG‐CoA and NADPH. HMGCR activity was determined by monitoring the rate of NADPH oxidation, measured as the decrease in absorbance at 340 nm over a 20‐min incubation period at 37°C. Results were normalized to tissue weight and expressed as U/g.

### 4.27 Human Sample

Left ventricular papillary muscle samples were obtained from patients undergoing cardiac surgery at the First Affiliated Hospital, Zhejiang University School of Medicine. The hyperlipidemia cohort comprised individuals with preserved cardiac function and confirmed dyslipidemia (elevated serum lipid levels in two independent measurements ≥4 weeks apart, including preoperative fasting lipid profiling). Controls were normolipidemic subjects without lipid‐lowering medication use. General inclusion criteria required age >18 years, with no exclusions based on gender, ethnicity, or race. Human tissue collection complied with the Declaration of Helsinki and was approved by the Institutional Review Board of the First Affiliated Hospital, Zhejiang University (Ethics Approval No. 2016‐198‐1). Written informed consent was obtained from all participants. Detailed clinical characteristics are provided in Table S4.

### 4.28 Statistical Analysis

Statistical analyses were performed using R (v4.0.3). Normality and homogeneity of variance were assessed using Shapiro‐Wilk and Bartlett tests, respectively. For comparisons between two groups, two‐tailed unpaired Student's t‐tests were applied. For multi‐group comparisons, two‐way ANOVA with Tukey‐Kramer post hoc correction was used to account for multiple comparisons. Data are presented as mean ± SEM unless otherwise specified, with statistical significance defined as *p* < 0.05. All analyses were conducted by investigators blinded to experimental conditions.

## Funding

This work was supported by grants from the National Natural Science Foundation of China (82400278 to Y.C., U21A20337 to X.G., 82370428, and 82070409 to J.Y., 82403935 to X.L.), Zhejiang Provincial Natural Science Foundation (Z25H020003 to J.Y., ZCLQN25H0202 to Y.C., LQ24H160024 to X.L., LY23H020003 to J.H.).

## Ethics Statement

Human tissue collection complied with the Declaration of Helsinki and was approved by the Institutional Review Board of the First Affiliated Hospital, Zhejiang University (Ethics Approval No. 2016‐198‐1). Written informed consent was obtained from all participants. All animal procedures were conducted in accordance with the national guidelines for experimental animal welfare and were approved by the Committee for Experimental Animal Science of Zhejiang University (Approval No: ZJU20200102).

## Conflicts of Interest

The authors declare no conflicts of interest.

## Supporting information




**Supporting File**: advs74567‐sup‐0001‐SuppMat.docx.

## Data Availability

The data underlying this article will be shared on reasonable request to the corresponding author.
