# Supplementary material for: Farnesyltransferase Deficiency in Cardiomyocytes Initiates Senescence and Contributes to Cardiac Fibrosis
Source: Adv Sci (Weinh). 2026 Feb 26;13(26):e11530. doi: 10.1002/advs.202511530 (PMC13159113; doi:10.1002/advs.202511530)
Supplement: Supplementary file 1 — Supporting File: advs74567‐sup‐0001‐SuppMat.docx. [file ADVS-13-e11530-s001.docx]

**Supplementary Table 1: siRNA Sequences for Targeted Gene Silencing**

| siRNA | Species | Sequence (5’→3’) |
| --- | --- | --- |
| *Ctcf* siRNA | Mouse | Fwd: GUGCAAUUGAGAACAUUAUTT  Rev: AUAAUGUUCUCAAUUGCACTT |
| *Pparg* siRNA | Mouse | Fwd: CCAUCCGAUUGAAGCUUAUTT  Rev: AUAAGCUUCAAUCGGAUGGTT |
| *Srebf1* siRNA | Mouse | Fwd: CCCGCUGCUUUAAAGAUGUTT  Rev: ACAUCUUUAAAGCAGCGGGTT |
| *Srebf2* siRNA | Mouse | Fwd: GUCCCAUUAUCUACCUUCUTT  Rev: AGAAGGUAGAUAAUGGGACTT |
| *Tgfb2* siRNA | Mouse | Fwd: GACCCUACUUCAGAAUCGUTT  Rev: ACGAUUCUGAAGUAGGGUCTT |
| *Gdf15* siRNA | Mouse | Fwd: CUCAGUCCAGAGGUGAGAUTT  Rev: AUCUCACCUCUGGACUGAGTT |
| *Bcl2* siRNA | Rat | Fwd: GUGGGAUACUGGAGAUGAATT  Rev: UUCAUCUCCAGUAUCCCACTT |
| *Hsp70* siRNA | Rat | Fwd: GAUUGGACUACGCUUGACUTT  Rev: AGUCAAGCGUAGUCCAAUCTT |

**Supplementary Table 2: Primer Sequences for RT-qPCR**

| Gene | Species | Sequence (5’→3’) |
| --- | --- | --- |
| *Col1A1* | Mouse | Fwd: TCCTGACGCATGGCCAAGAAGACA Rev: TCCGGGCAGAAAGCACAGCACTC |
| *Col3A1* | Mouse | Fwd: CTGTAACATGGAAACTGGGGAAA Rev: CCATAGCTGAACTGAAAACCACC |
| *Creb* | Mouse | Fwd: GAAGAGGAGACTTCAGCCC Rev: TAATGGCAATGTACTGCCCA |
| *Ctcf* | Mouse | Fwd: ATGATCCCAACTTTGTCCCT Rev: GTCTTGCCATTGTGTTCCG |
| *E2F4* | Mouse | Fwd: CTGAGGACGTCCAGAACAG Rev: CAAGGAGGGTATCTCCAGC |
| *Esr1* | Mouse | Fwd: CCTCTGGCTACCATTATGGG Rev: AGTCATTGTGTCCTTGAATGC |
| *Ets1* | Mouse | Fwd: ACTTCATCAGCTACGGTATCG Rev: AGGACTCTGTGATGAAGCTG |
| *Fli1* | Mouse | Fwd: TCACTGCTGGCCTATAACAC Rev: AGAGTCATAAGAAGGGTCTTCC |
| *Fnta* | Mouse | Fwd: AGCACTAGAGTTATGTGAGATCC Rev: AGGGACCGTCCAATATACCT |
| *Fntb* | Mouse | Fwd: ATTTGTACTCCCTAAAGCAACC Rev: CACAGTATGCACTCCTTACG |
| *Gata1* | Mouse | Fwd: GCATCTTCTTCCACTTCCC Rev: GATACACCTGAAAGACTGGG |
| *Gdf15* | Mouse | Fwd: AGTGTCCCCACCTGTATCG Rev: TGTCCTGTGCATAAGAACCA |
| *Il6* | Mouse | Fwd: GAGAAAAGAGTTGTGCAATG Rev: ATTTTCAATAGGCAAATTTC |
|  |  |  |
| Gene | Species | Sequence (5’→3’) |
| *Mmp2* | Mouse | Fwd: TTCAACGGTCGGGAATACAG Rev: AACTTGCAGGGCTGTCCATC |
| *Myc* | Mouse | Fwd: CTGTACCTCGTCCGATTCC Rev: GCTCTTCTTCAGAGTCGCT |
| *P15* | Mouse | Fwd: AGATCCCAACGCCCTGAAC Rev: CCCATCATCATGACCTGGATT |
| *P16* | Mouse | Fwd: CAGATTCGAACTGCGAGGA Rev: CAGCGGAACACAAAGAGCA |
| *P19* | Mouse | Fwd: GAGAGGGTTTTCTTGGTGA Rev: AGAAGAGCTGCTACGTGA |
| *P21* | Mouse | Fwd: ATGTCCAATCCTGGTGATGT Rev: TGCAGCAGGGCAGAGGAAGT |
| *P53* | Mouse | Fwd: GAGCTCCCTCTGAGCCAGGA Rev: TGGGCCTTCAAAAAACTCCTCA |
| *Pparg* | Mouse | Fwd: GATGTCTCACAATGCCATCAG Rev: ATATCACTGGAGATCTCCGC |
| *Rad21* | Mouse | Fwd: CAGAGTAGAAGAGATAACCATGAG Rev: ATTCCGAAGTCACCAAAGTC |
| *Srebf1* | Mouse | Fwd: GTGAACATCTCCTAGAGCGA Rev: CTGAGAACTCCCTGTCTCC |
| *Srebf2* | Mouse | Fwd: ATGCCAAGATGCACAAGTC Rev: GCAGCTTGTGATTGACCTG |
| *Tead4* | Mouse | Fwd: CGGAGAACATGATTATCACCT Rev: CATACTCCGTCTCAACTTTCTC |
| *Tgfb1* | Mouse | Fwd: ACCAAGGAGACGGAATACAG Rev: CGTTGATTTCCACGTGGAG |
| *Tgfb2* | Mouse | Fwd: CGGATTGAACTGTATCAGATCC Rev: TCGAAGGAGAGCCATTCAC |
| Gene | Species | Sequence (5’→3’) |
| *Tgfb3* | Mouse | Fwd: ACTATGCCAACTTCTGCTC Rev: TGTATAGTCCAAGCACCGT |
| *Timp1* | Mouse | Fwd: ATTCAAGGCTGTGGGAAATG Rev: CTCAGAGTACGCCAGGGAAC |
| *Timp2* | Mouse | Fwd: CACAGACTTCAGCGAATGGA Rev: CTTGGGAAGCTTGAGAGTGG |
| *Tnf* | Mouse | Fwd: TACTGAACTTCGGGGTGATTGGTCC Rev: CAGCCTTGTCCCTTGAAGAGAACC |
| *Cdkn2A* | Rat | Fwd: TGGTCACTGTGAGGATTCG Rev: CATCATCACCTGGTCCAGG |
| *Cdkn2B* | Rat | Fwd: CTAATGGTGCTGCACAAGG Rev: ATTCTTCACCTCGCAATATCAC |
| *Cdkn2D* | Rat | Fwd: GGCCGGGGAATAACCATAGA Rev: CAAGCCCAATTCAGGTCAGG |
| *Cdkn1A* | Rat | Fwd: CTGAGAGGCCTGAAGACTC Rev: GTGATAGAAATCTGTTAGGCTGG |
| *Tp53* | Rat | Fwd: ATGATATTCTGCCCACCAC Rev: ACAACTCTGCAACATCCTG |
| *Tgfb2* | Rat | Fwd: CTATTGCTTTAGGAATGTGCAG Rev: CATTTCCATCCAAGATCCCT |
| *Gdf15* | Rat | Fwd: TCTGAGTCCCAACTCAACC Rev: CACCTCTGGACTGAGTATCC |
| *Bcl2* | Rat | Fwd: TGACTGAGTACCTGAACCG Rev: TAGTTCCACAAAGGCATCC |
| *Hspa1A* | Rat | Fwd: CAAGGCCAACAAGATCACC Rev: CTCGATCTCCTCCTTGCTC |
| *Fnta* | Rat | Fwd: CCGATATCCTTAATCAGGATGC Rev: CCCAAAGTCGAAACTCCTG |

**Supplementary Table 3: Primary Antibodies Used for Western Blot and Immunofluorescent Staining**

| **Antibody** | **Company** | **Catalog Number** |
| --- | --- | --- |
| anti-α-SMA antibody | Abcam | Cat# ab5694 |
| anti-α-Actinin antibody | Abcam | Cat# ab9465 |
| anti-ATM antibody | Abcam | Cat# ab199726 |
| anti-BAX antibody | Proteintech | Cat# 50599-2-Ig |
| anti-BCL-2 antibody | Proteintech | Cat# 26593-1-AP |
| anti-Caspase3 antibody | Cell Signaling Technology | Cat# 9662 |
| anti-Caspase3 antibody | Abclonal | Cat# A19664 |
| anti-β-Tubulin antibody | Proteintech | Cat# 10094-1-AP |
| anti-CHK1 antibody | Abclonal | Cat# A7653 |
| anti-CHK2 antibody | Abclonal | Cat# A2145 |
| anti-FNTA antibody | Abcam | Cat# ab109738 |
| anti-FNTB antibody | Proteintech | Cat# 66783-1-Ig |
| anti-FNTB antibody | Proteintech | Cat#11507-1-AP |
| anti-GAPDH antibody | Abcam | Cat# ab181602 |
| anti-GATA4 antibody | Proteintech | Cat#19530-1-AP |
| anti-GDF15 antibody | Proteintech | Cat# 27455-1-AP |
| anti-GDF15 antibody | Abcam | Cat# ab39999 |
| anti-H_2_AX (Ser139) antibody | GeneTex | Cat# GTX127340 |
| anti-HSP70 antibody | Proteintech | Cat# 10995-1-AP |
| anti-HSP70 antibody | Abclonal | Cat# A21180 |
| anti-p-ATM (Ser1981) antibody | R&D | Cat# AF1655-SP |
| anti-p-CHK1 (Ser317) antibody | Cell Signaling Technology | Cat# 12302 |
| anti-p-CHK2 (Thr68) antibody | Invitrogen | Cat# MA5-27988 |
| anti-PrelaminA antibody | Millipore | Cat# MABT345 |
| anti-TGF-β2 antibody | Proteintech | Cat# 19999-1-AP |
| anti-TGF-β2 antibody | Abclonal | Cat# A25496 |
| Antibody | Company | Catalog Number |
| anti-TIMP2 antibody | Abclonal | Cat# A1588 |
| anti-p-RIPK1(Ser166) antibody | Cell Signaling Technology | Cat# 31122 |
| anti-p-MLKL(Ser345) antibody | Cell Signaling Technology | Cat# 37333 |
| anti-SREBF2 antibody | Abclonal | Cat# A13049 |
| anti-H3K9me3 antibody | Abclonal | Cat# A2360 |
| anti-HMGCR antibody | Proteintech | Cat# 13533-1-AP |
| anti-MVK antibody | Huabo | Cat# HA601151 |
| anti-FDPS antibody | Proteintech | Cat# 16129-1-AP |
| anti-GGPS1 antibody | Santa Cruz | Cat# sc-271680 |

**Supplementary Table 4：Clinical Characteristics of Control and Hyperlipidemia Cohorts**

| Group | Age | Gender | Primary Diagnosis | CAD | HTN | DM | Fatty Liver | Other  Metabolic Diseases | Statins | BB | ACEI/ARB | CCB | SGLT2 Inhibitors | Other Drugs | EF |
| --- | --- | --- | --- | --- | --- | --- | --- | --- | --- | --- | --- | --- | --- | --- | --- |
| Control | 55 | Male | MS, AS, AI | - | - | - | - | - | - | Metoprolol | - | Diltiazem | - | - | 0.639 |
|  | 52 | Male | MI, TI | - | - | - | - | - | - | - | - | - | - | - | 0.66 |
|  | 53 | Male | MS, MI | - | - | - | - | - | - | - | - | - | - | - | 0.71 |
|  | 67 | Female | MS, TI | - | - | - | - | - | - | - | - | - | - | - | 0.56 |
|  | 50 | Female | MS, MI | - | - | - | - | - | - | - | - | - | - | Digoxin | 0.54 |
|  | 56 | Female | MS | - | - | - | - | - | - | Metoprolol | - | - | - | Digoxin | 0.65 |
| Hyperlipidemia | 44 | Female | MI, TI, AI | - | - | - | - | - | - | - | - | - | - | Digoxin | 0.61 |
|  | 58 | Male | MS, MI | Yes | - | - | - | - | Rosuvastatin | Metoprolol | - | - | - | - | 0.706 |
|  | 66 | Male | Infective Endocarditis | - | Yes | - | - | - | Atorvastatin | Metoprolol | - | - | - | Kardaleon | 0.675 |
|  | 59 | Female | MS, MI | - | Yes | - | - | Hyperuricemia | - | - | - | - | - | - | 0.59 |
|  | 55 | Male | MS, TI | - | - | - | - | - | Atorvastatin | Metoprolol | - | - | - | Kardaleon | 0.6 |
|  | 63 | Male | AI | - | Yes | - | - | - | - | - | - | Cinnarizine | - | - | 0.76 |

Abbreviations: MS: Mitral Stenosis, MI: Mitral Insufficiency, TI: Tricuspid Insufficiency, AS: Aortic Stenosis, AI: Aortic Insufficiency; CAD: Coronary Artery Disease, HTN: Hypertension, DM: Diabetes Mellitus; BB: Beta-Blockers, CCB: Calcium Channel Blockers, SGLT2: Sodium-Glucose Cotransporter-2

**Figure S1**


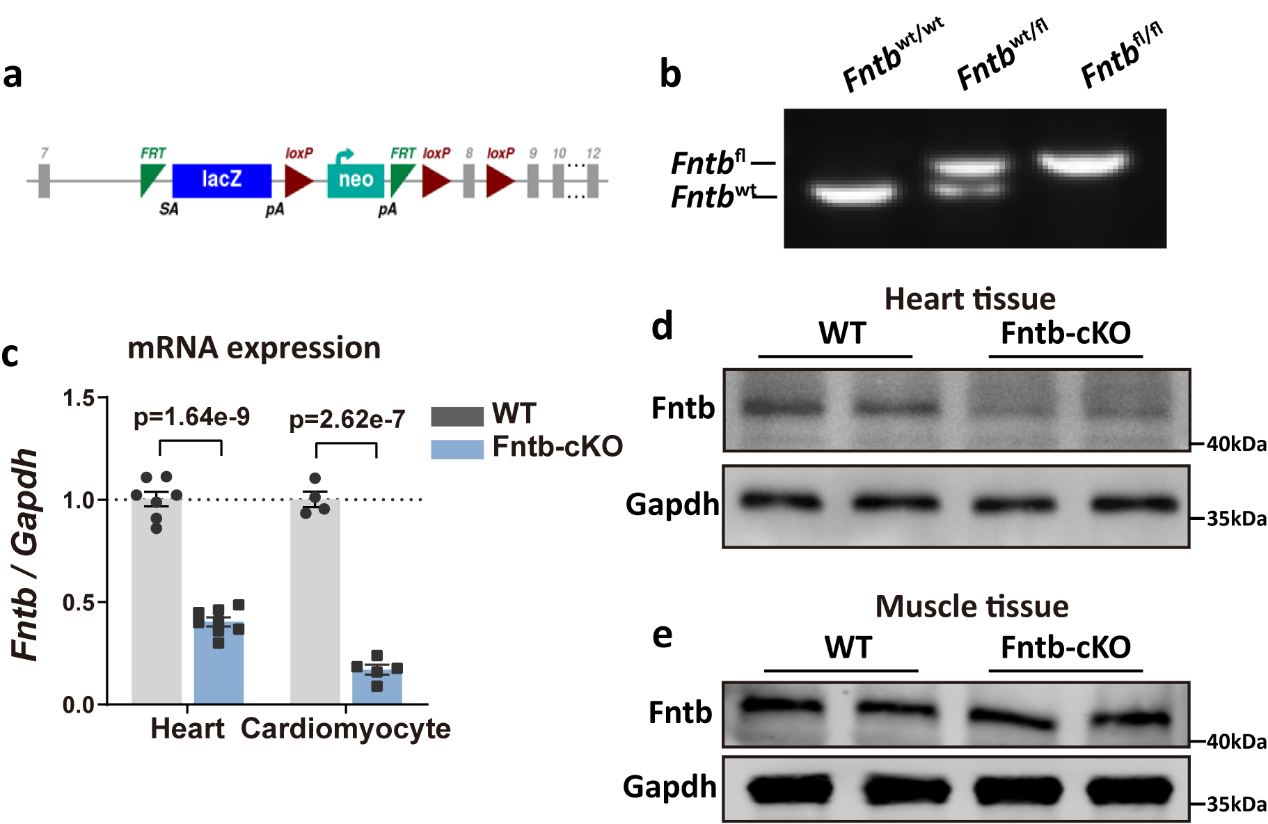


**Figure S1. Generation and Characterization of cardiomyocyte-specific *Fntb* knockout (*Fntb* cKO) Mice**

**(a)** Targeting strategy schematic for constructing *Fntb* floxed (*Fntb* ^fl/fl^) mice with loxP sites (triangles) flanking exon 8. Key restriction enzyme recognition sites are annotated. **(b)** Representative agarose gel electrophoresis showing PCR-amplified fragments of wild-type (*Fntb* ^wt^, 455 bp) and floxed (*Fntb* ^fl^, 592 bp) alleles. Genotypes of donor mice are indicated above each lane. **(c)** Validation of *Fntb* knockout efficiency by RT-qPCR. mRNA levels of *Fntb* in cardiac tissues (left) and isolated cardiomyocytes (right) from *Fntb* cKO mice showed significant reduction compared to controls. n=4-7/group. **(d)** Western blot analysis confirming ablation of Fntb protein expression in cardiac tissue of *Fntb* cKO mice. Gapdh served as loading control. **(e)** Tissue-specific knockout validation showing intact *Fntb* expression in skeletal muscle of cKO mice. Gapdh served as loading control. Data presented as mean ± SEM; Statistical significance was determined by a two-tailed unpaired t-test.

**Figure S2**


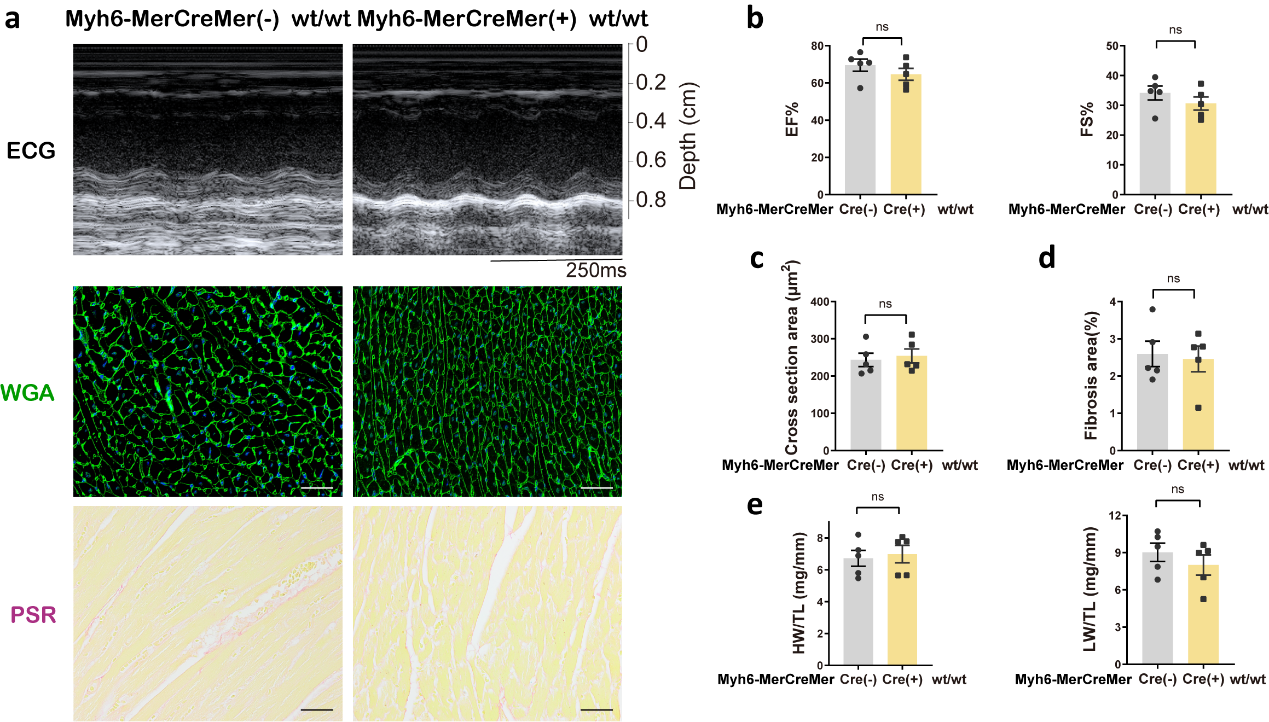


**Figure S2. Cre Recombinase Expression Does Not Induce Cardiac Remodeling**

**(a)** Assessment of Myh6 MerCreMer (+) *Fntb* wt/wt mice two weeks after tamoxifen induction. Echocardiographic (ECG) analysis (top) reveals preserved systolic function; WGA (middle) and PSR (bottom) staining demonstrate comparable cardiomyocyte cross-sectional areas and interstitial collagen deposition relative to baseline; **(b)** Quantification of ejection fraction (EF) and fractional shortening (FS) shows no intergroup differences (n=5/group); **(c)** Cardiomyocyte cross-sectional area analysis from WGA staining confirms absence of hypertrophy (n=5/group); **(d)** Interstitial fibrosis quantification by PSR staining reveals no significant fibrotic remodeling (n=5/group); **(e)** Heart weight (HW) and lung weight (LW) normalized to tibial length (TL) remain unchanged in MerCreMer(+) mice (n=5/group). Data presented as mean ± SEM; Statistical significance was determined by a two-tailed unpaired t-test.

**Figure S3**


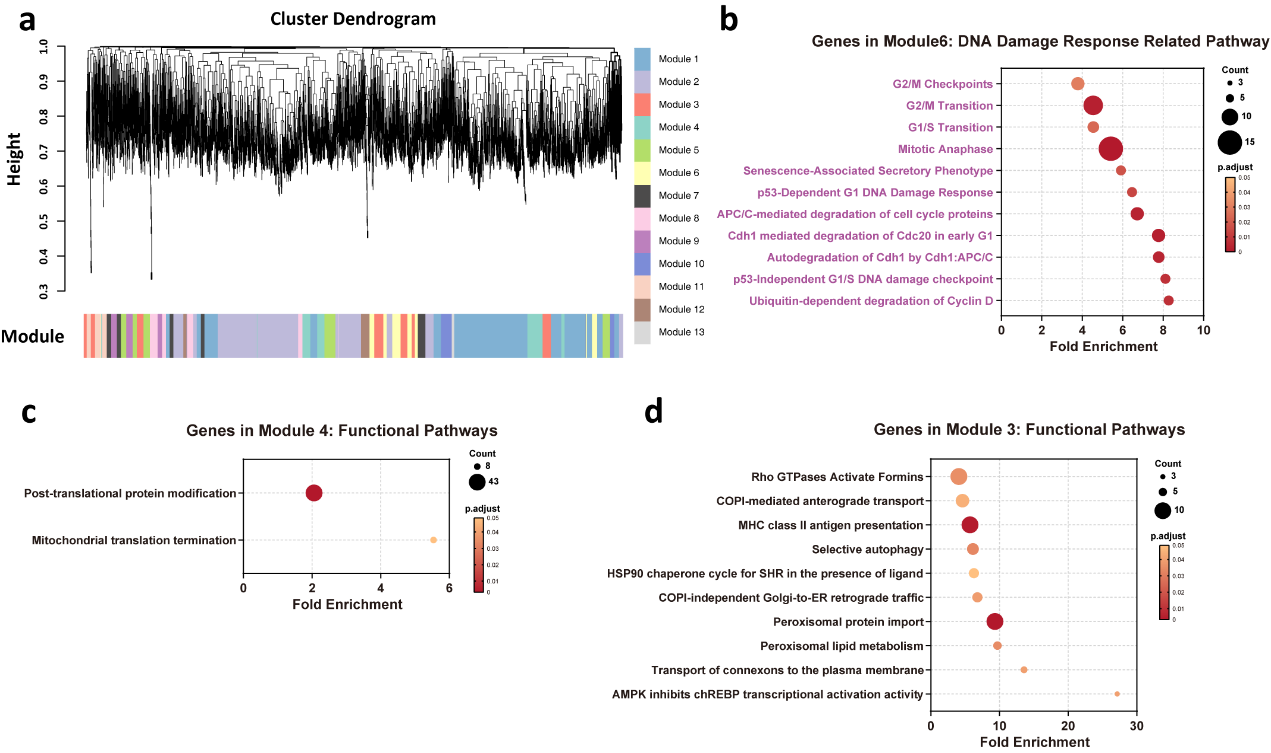


**Figure S3. Weighted Gene Co-Expression Network Analysis (WGCNA) of FNTB-cKO Adult Mouse Primary Cardiomyocytes**

(a) Scale-free hierarchical clustering of co-expressed genes identifies 13 modules. The upper panel displays a dendrogram illustrating gene clustering patterns (each leaf representing one gene), while the lower panel shows module assignments color-coded by size. **(b)** Module 6 enrichment analysis identified 11 DNA damage response-related pathways meeting statistical significance (adjusted P < 0.05). **(c)** Significantly enriched cellular pathways associated with Module 4; **(d)** Significantly enriched cellular pathways associated with Module 3.

**Figure S4**


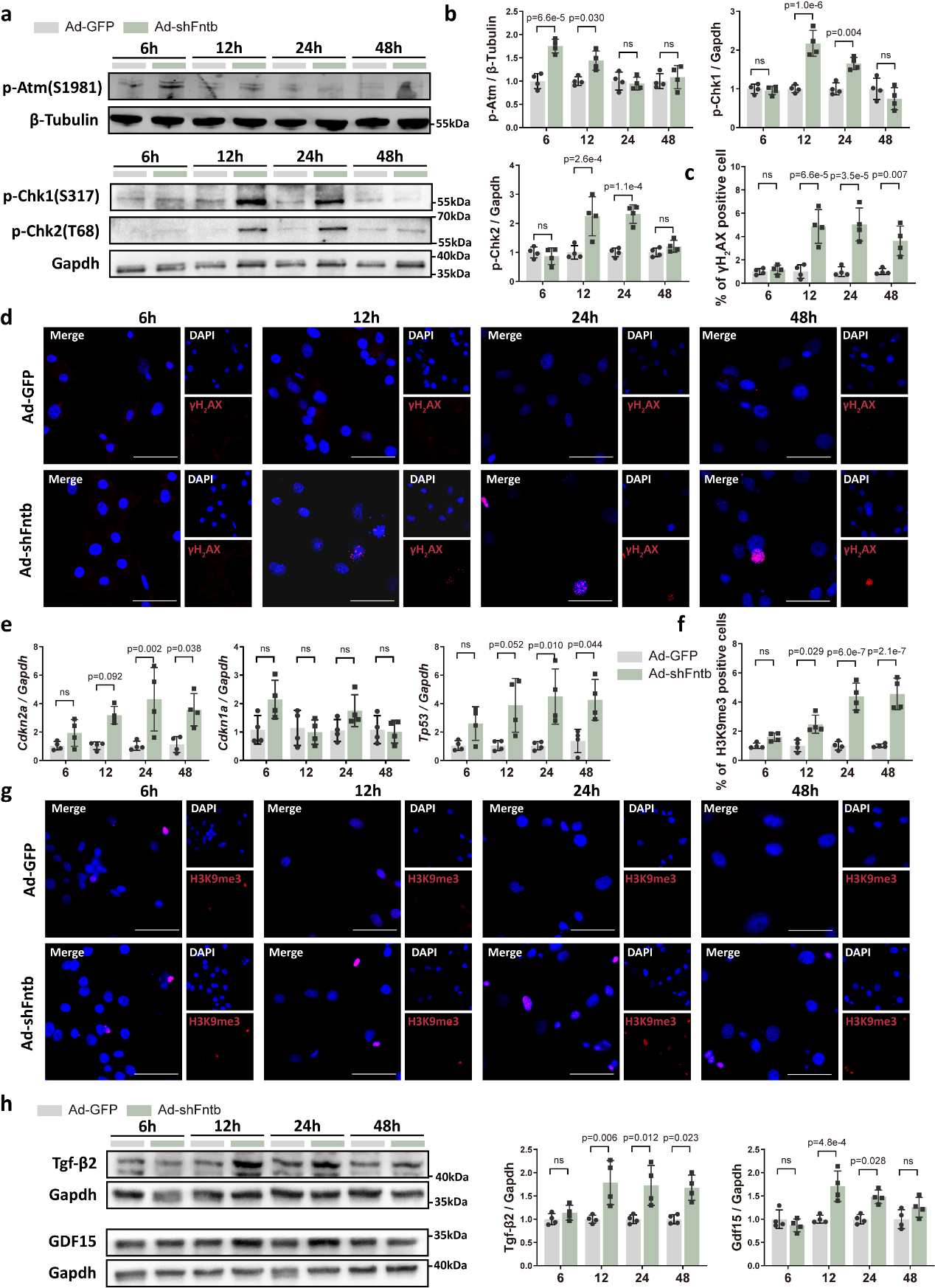


**Figure S4: *Fntb* Knockdown Triggers Temporal Progression of DNA Damage Response, Cellular Senescence, and SASP in neonatal rat cardiomyocytes (NRCMs)**

**(a-b)** Representative Western blot **(a)** and quantitative analysis **(b)** of phospho-Atm (Ser1981), phospho-Chk1 (Ser317), and phospho-Chk2 (Thr68) levels in NRCMs infected with Ad-GFP or Ad-shFntb for 6, 12, 24, or 48 hours (n=4/group); **(c-d)** Immunofluorescence detection of γH_2_AX foci (red) **(d)** and quantitative analysis of γH_2_AX -positive cells **(c)** in NRCMs across indicated time points (n=4/group). Scale bar: 50μm; (e) RT-qPCR analysis of mRNA expression levels for cyclin-dependent kinase inhibitors *Cdkn2a*, *Cdkn1a*, and *Tp53* in NRCMs (n=4/group); **(f-g)** Immunofluorescence detection of H3K9me3 foci (red) **(g)** and quantitative analysis of H3K9me3-positive nuclei **(f)**, indicating senescence-associated heterochromatic foci (SAHF) formation (n=4/group). Scale bar: 50μm; **(h)** Representative Western blot and quantitative analysis of senescence-associated secretory phenotype (SASP) components Tgf-β2 and Gdf15 protein levels in NRCMs following Fntb knockdown (n=4/group). All data represent mean ± SEM. ns: not significant. Multi-group comparisons were analyzed using two-way ANOVA with Tukey-Kramer post hoc test.

**Figure S5**


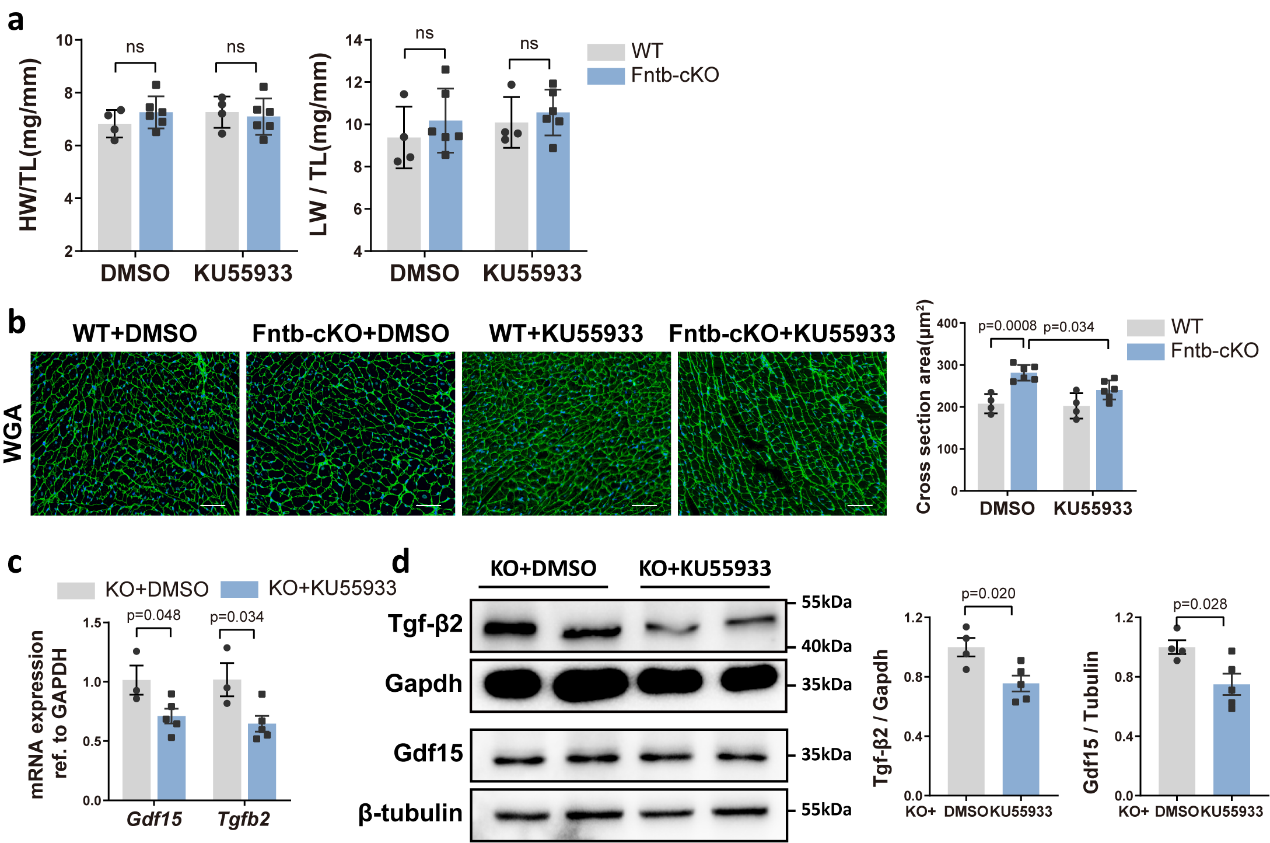


**Figure S5. DNA Damage Response Inhibition Attenuates *Fntb* Knockout-Induced Cardiomyocyte Hypertrophy and SASP Factor Induction**

**(a)** HW and lung weight (LW) alterations across groups (n=4-6/group); **(b)** WGA staining of cardiac sections demonstrates cardiomyocyte cross-sectional area changes, with quantitative analysis confirming hypertrophy attenuation in KU55933-treated cohorts (n=4-6/group), scale bar: 50 μm; **(c)** *Gdf15* and *Tgfb2* mRNA levels in KU55933-treated *Fntb*-cKO AMCMs were analyzed by RT-qPCR (n=3-5/group); **(d)** Western blot analysis of Gdf15 and Tgfb2 protein levels in KU55933-treated *Fntb*-cKO AMCMs (n=4-5/group). All data expressed as mean ± SEM. Two-group comparisons were performed with unpaired two-tailed Student’s t-test. Multi-group comparisons analyzed by two-way ANOVA followed by Tukey-Kramer post hoc test.

**Figure S6**


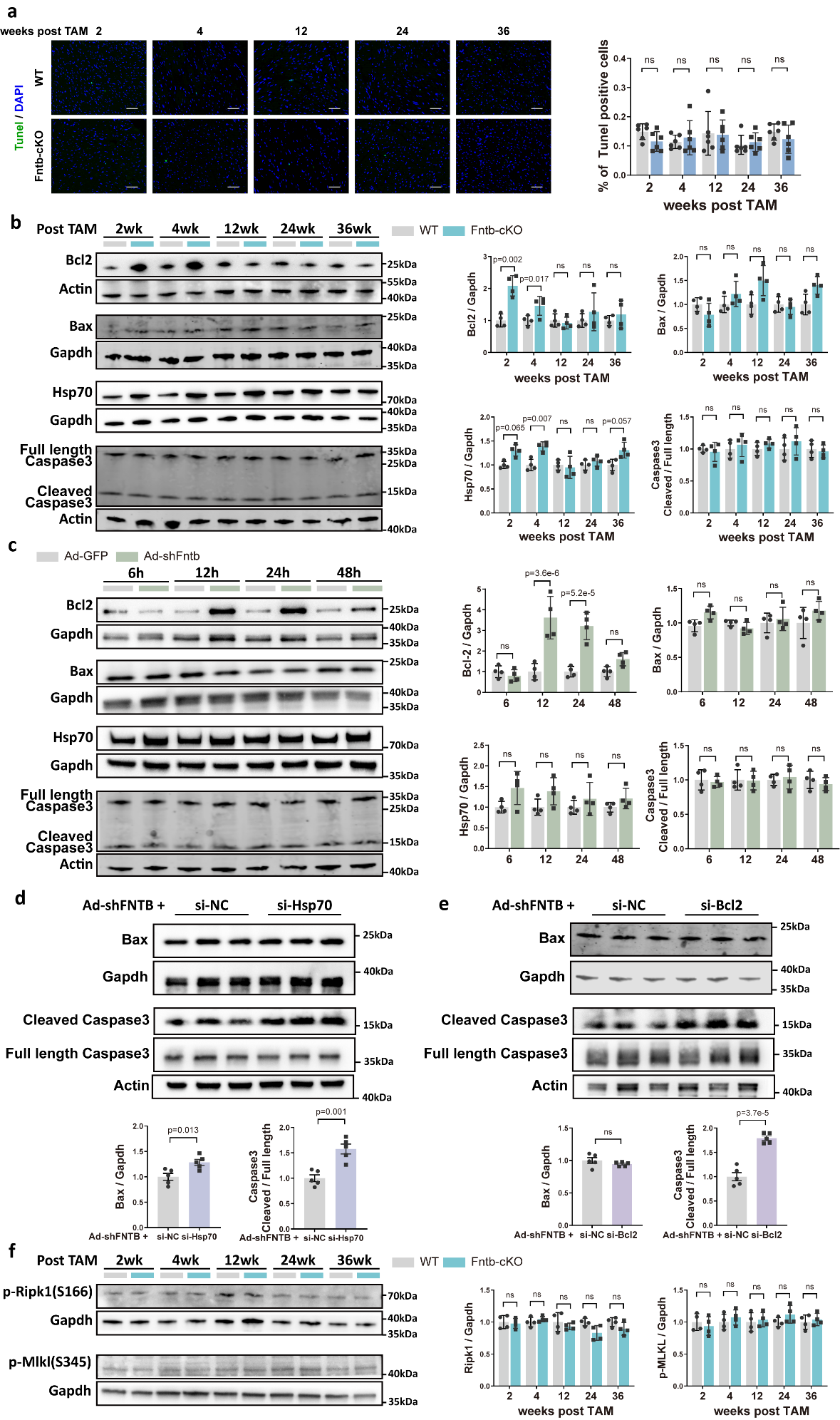


**Figure S6. Fntb Deficiency is Not Associated with Cardiomyocyte Apoptosis or Necroptosis**

**(a)** Representative TUNEL staining (left) and quantification (right) of TUNEL-positive nuclei in heart sections from WT and *Fntb*-cKO mice at 2, 4, 12, 24, and 36 weeks post-tamoxifen (TAM) induction (n=6/group). Scale bars: 50 μm; **(b)** Western blot analysis and quantification of Bcl2, Bax, Hsp70, and Caspase-3 in AMCMs from WT and *Fntb*-cKO mice at indicated time points post-TAM (n=4/group); **(c)** Western blot analysis and quantification of Bcl2, Bax, Hsp70, and Caspase-3 in NRCMs infected with Ad-GFP or Ad-shFntb for 6, 12, 24, or 48 hours (n=4/group); **(d, e)** Western blot and quantitative analysis of Bax, cleaved Caspase-3 in Fntb-silenced NRCMs following siRNA-mediated knockdown of Hsp70 **(d)** or Bcl2 **(e)** (n=5/group); **(f)** Western blot analysis and quantification of necroptosis markers, including phospho-Ripk1 (Ser166) and phospho-Mlkl (Ser345), in AMCMs isolated from WT and Fntb-cKO mice at indicated time points post-TAM (n=4/group). All data represent mean ± SEM. ns: not significant. Multi-group comparisons were analyzed using two-way ANOVA with Tukey-Kramer post hoc test. Two-group comparisons were performed with unpaired two-tailed Student’s t-test.

**Figure S7**


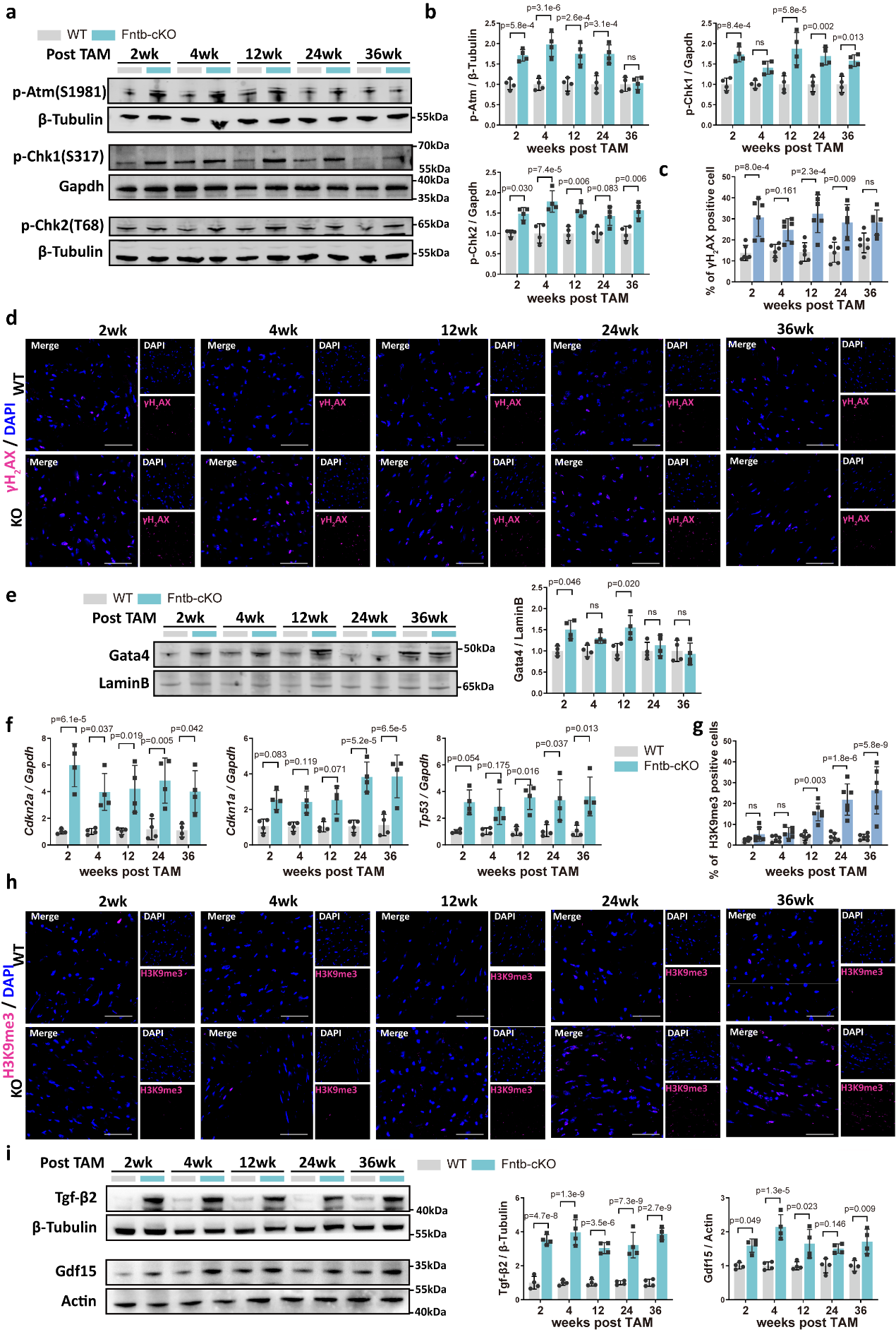


**Figure S7: Fntb Deficiency Triggers Temporal Progression of DNA Damage Response, Cellular Senescence, and SASP in Vivo**

**(a-b)** Representative Western blot **(a)** and quantitative analysis **(b)** of phospho-Atm (Ser1981), phospho-Chk1 (Ser317), and phospho-Chk2 (Thr68) levels in AMCMs isolated from WT and Fntb-cKO mice at 2, 4, 12, 24, or 36 weeks post-TAM induction (n=4/group); **(c-d)** Immunofluorescence detection of γH_2_AX foci (pink) **(d)** and quantitative analysis of γH_2_AX-positive cells **(c)** in heart sections across indicated time points (n=6/group). Scale bars: 50μm; **(e)** Representative Western blot and quantitative analysis of Gata4 in AMCMs (n=4/group); **(f)** RT-qPCR analysis of mRNA expression levels for *Cdkn2a*, *Cdkn1a*, and *Tp53* in AMCMs (n=4/group); **(g-h)** Immunofluorescence detection of H3K9me3 foci (pink) **(h)** and quantitative analysis of H3K9me3-positive nuclei **(g)** in heart sections, indicating SAHF formation (n=6/group). Scale bars: 50μm; **(i)** Representative Western blot and quantitative analysis of SASP components Tgf-β2 and Gdf15 protein levels in AMCMs following Fntb knockout (n=6/group). Data represent mean ± SEM. ns: not significant. Multi-group comparisons were analyzed using two-way ANOVA with Tukey-Kramer post hoc test.

**Figure S8**


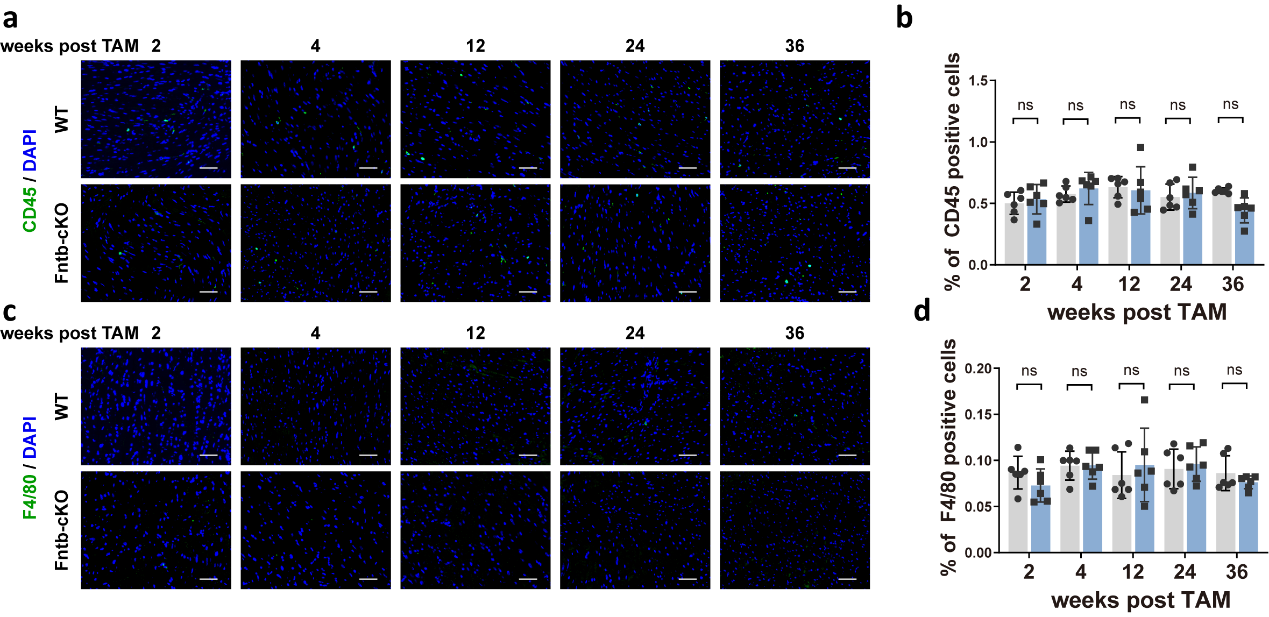


**Figure S8: Fntb Deficiency Does Not Induce Significant Inflammatory Cell Infiltration in the Heart**

**(a-b)** Representative immunofluorescence images **(a)** and quantitative analysis **(b)** of CD45-positive leukocytes (green) in heart sections from WT and *Fntb*-cKO mice at 2, 4, 12, 24, and 36 weeks post-TAM induction (n=6/group); **(c-d)** Representative immunofluorescence images **(c)** and quantitative analysis **(d)** of F4/80-positive macrophages (green) in heart sections from WT and Fntb-cKO mice across the indicated time points (n=6/group). Scale bars: 50μm. Data represent mean ± SEM. ns: not significant. Multi-group comparisons were analyzed using two-way ANOVA with Tukey-Kramer post hoc test.

**Figure S9**


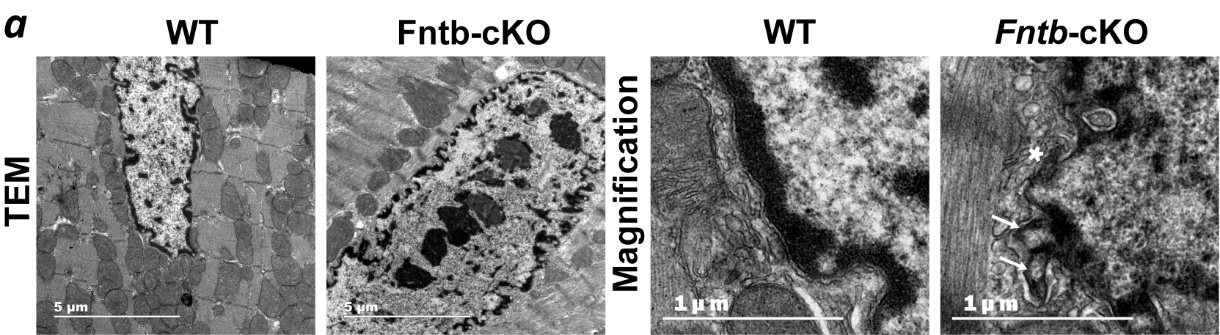


**Figure S9: Fntb Knockout Disrupts Nuclear Membrane Integrity in Cardiomyocytes**

**(a)** Transmission electron microscopy (TEM) images reveal nuclear envelope defects in *Fntb*-cKO cardiomyocytes. The left panel displays irregular nuclear contours with serrated membranes, while the right panel shows discontinuous nuclear membranes (*) and irregular nuclear envelope protrusions (arrows) at higher magnification. Scale bars: 2 μm (left), 500 nm (right).

**Figure S10**


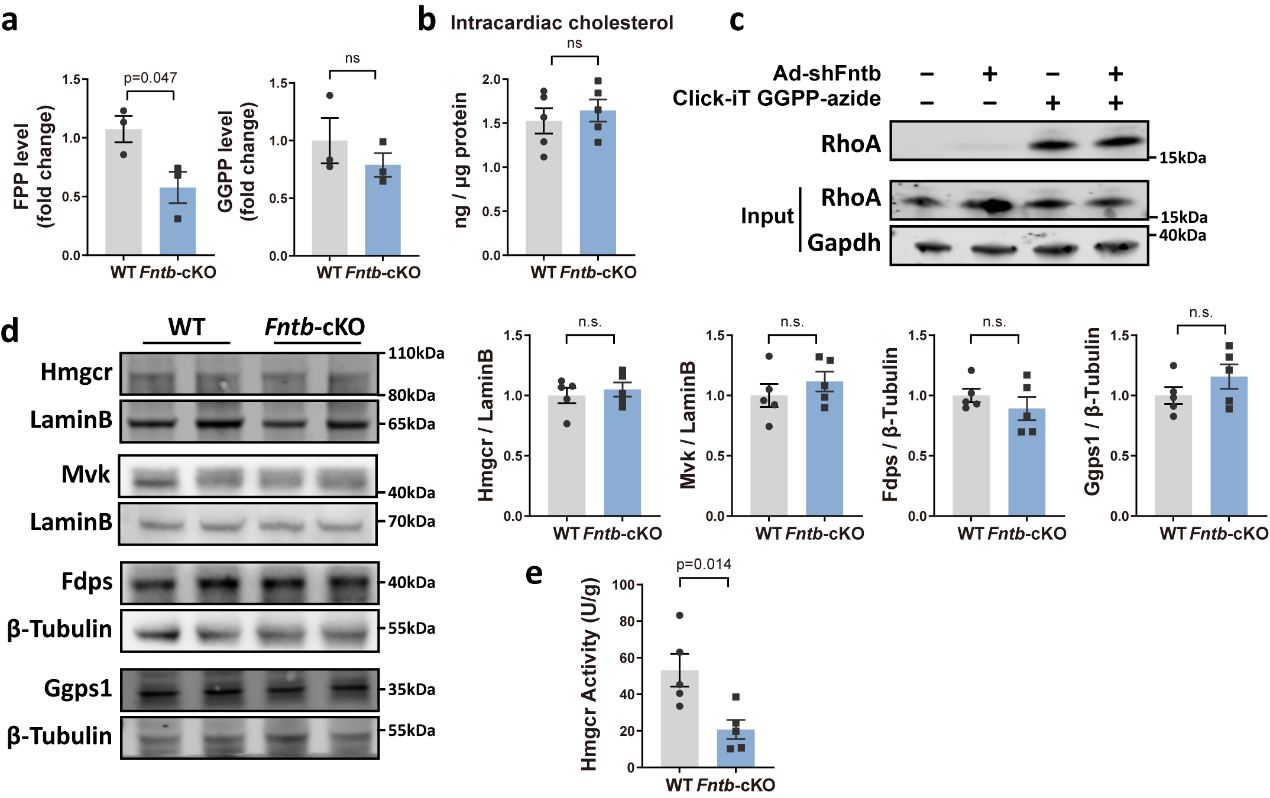


**Figure S10. Impact of *Fntb* Knockout on Cardiac Mevalonate Pathway Metabolites and Protein Geranylgeranylation**

**(a)** HPLC-MS/MS quantification of farnesyl pyrophosphate (FPP) and geranylgeranyl pyrophosphate (GGPP) levels in cardiac tissues of WT and *Fntb*-cKO mice (n=3/group)；**(b)** Total cholesterol content in cardiac tissues (n=5/group); **(c)** Click chemistry-based assessment of RhoA geranylgeranylation in NRCMs following Fntb knockdown. Cells were metabolically labeled with Click-iT GGPP-azide, a synthetic analog incorporated into substrate proteins by GGTase I. Ad-shFntb does not reduce the incorporation of GGPP into RhoA, demonstrating that the diminished FPP pool does not impair downstream metabolic flux; **(d)** Representative immunoblots and protein quantification of key mevalonate pathway enzymes, including Hmgcr, Mvk, Fdps, Ggps1 protein level in *Fntb*-cKO hearts (n=5/group); **(e)** Enzymatic activity of HMGCR in heart tissues from WT and *Fntb*-cKO mice, determined by the rate of NADPH oxidation (n=5/group). All data represent mean ± SEM. Between-group comparisons analyzed by unpaired two-tailed Student’s t-test.

**Figure S11**


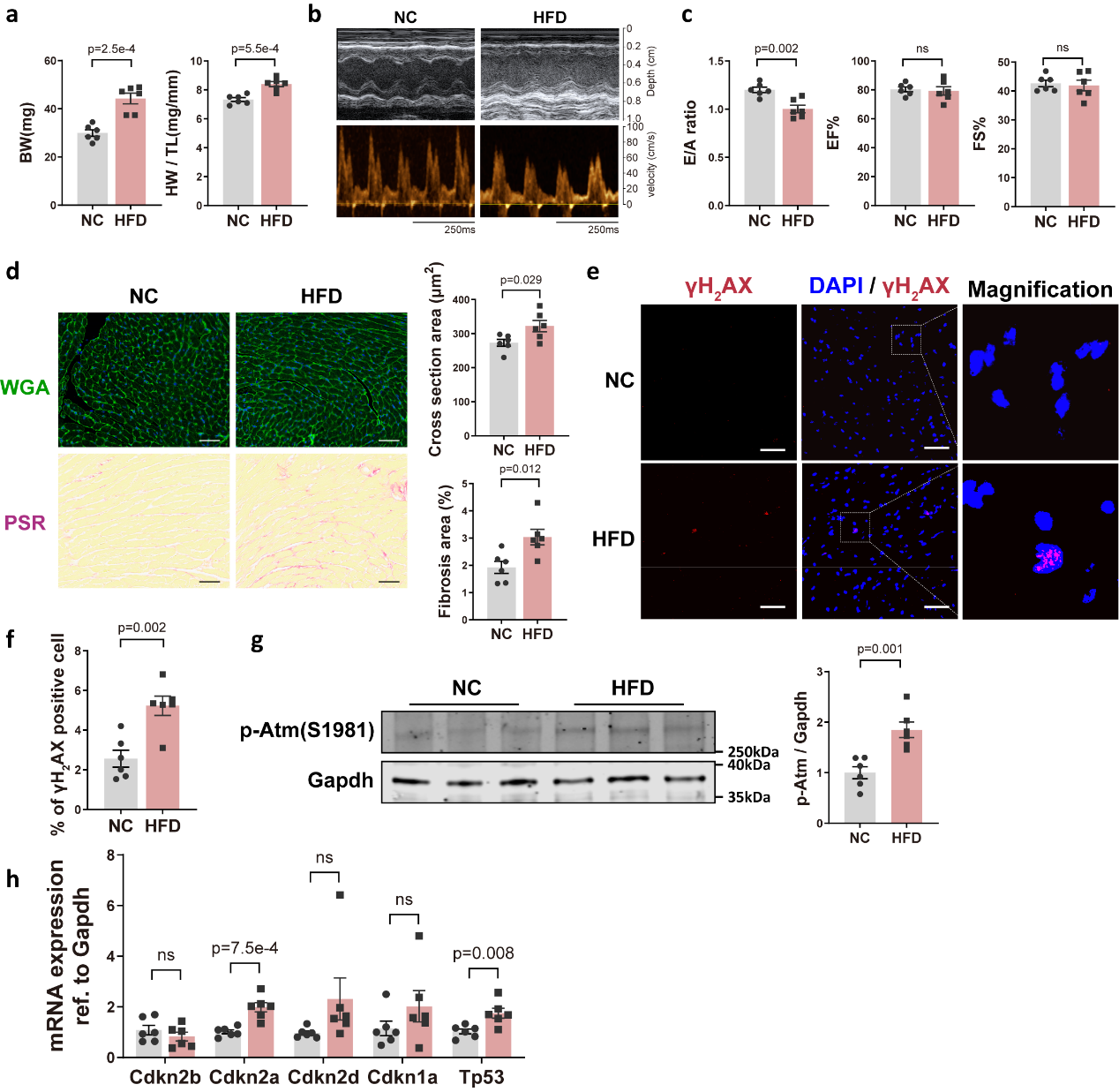


**Figure S11. High-Fat Diet (HFD) Induces Cardiac Hypertrophy, Diastolic Dysfunction, Fibrosis, and Cellular Senescence in Mice**

**(a)** Body weight (BW) and HW/TL in mice fed a normal chow (NC) or HFD for 20 weeks (n=6/group); **(b-c)** Representative M-mode and Doppler echocardiographic images **(b)** and quantitative analysis **(c)** of diastolic function (E/A ratio) and systolic function (EF% and FS%) (n=6/group); **(d)** Representative WGA staining (top) and PSR staining (bottom) of heart sections, with quantification of cardiomyocyte cross-sectional area and interstitial fibrosis area (n=6/group). Scale bars: 50μm; **(e, f)** Representative immunofluorescence images **(e)** and quantitative analysis **(f)** of γH_2_AX-positive nuclei (red) in heart sections, indicating DNA damage (n=6/group). Scale bars: 50μm; **(g)** Representative Western blot and quantification of phospho-Atm (Ser1981) protein levels in cardiac tissues (n=6/group); **(h)** RT-qPCR analysis of mRNA expression levels for senescence-associated genes, including *Cdkn2b*, *Cdkn2a*, *Cdkn2d*, *Cdkn1a*, and *Tp53*, in cardiac tissues (n=6/group). All data represent mean ± SEM. Between-group comparisons analyzed by unpaired two-tailed Student’s t-test.

**Figure S12**


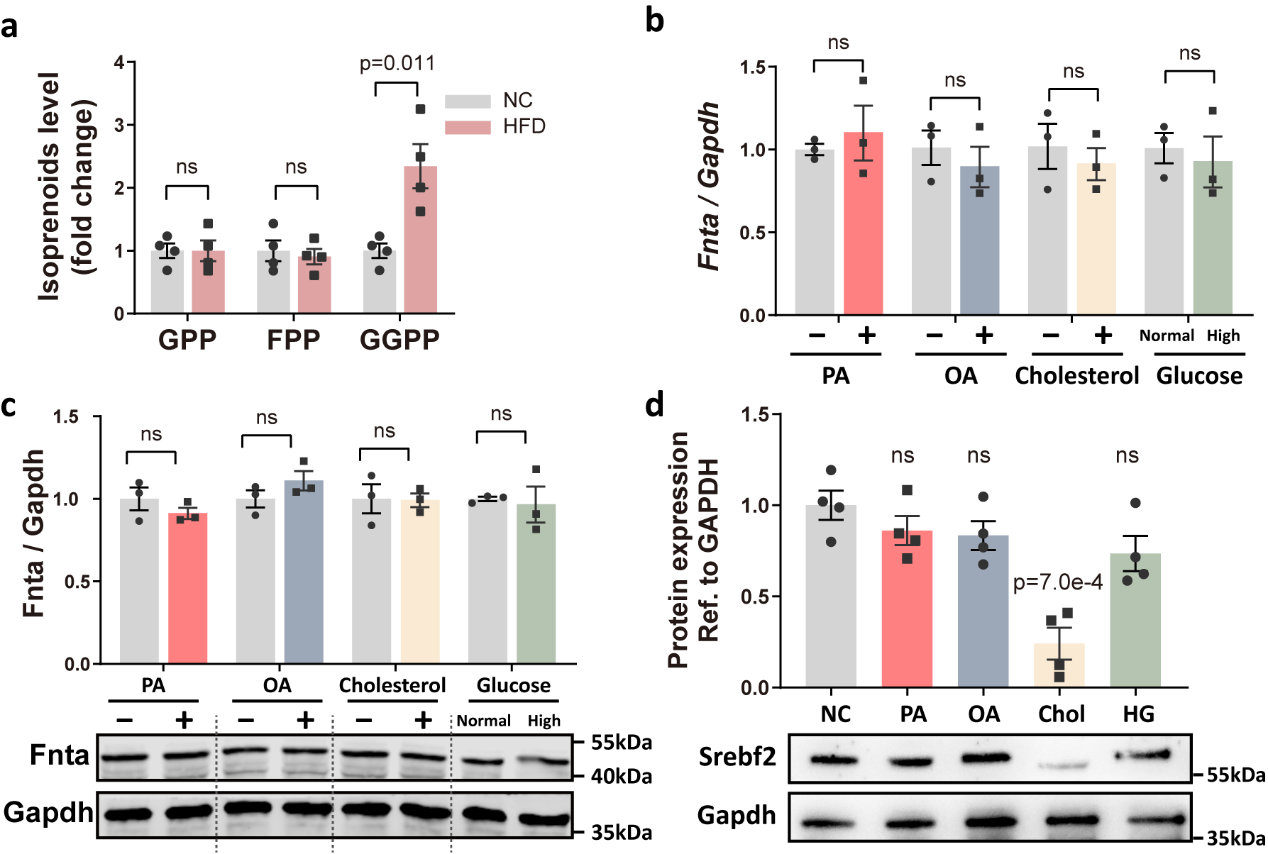


**Figure S12: Effects of Lipid Overload on Cardiac Isoprenoid Levels and the Expression of Fnta and Srebf2**
**(a)** HPLC-MS quantification of cardiac isoprenoid metabolites, including geranyl pyrophosphate (GPP), FPP, and GGPP, in hearts of mice fed NC or HFD for 20 weeks (n=4/group); **(b)** RT-qPCR analysis of *Fnta* mRNA levels in AMCMs treated with palmitic acid (PA, 500 μM), oleic acid (OA, 500 μM), cholesterol (100 μM), or high glucose (25 mM) (n=3/group); **(c)** Representative Western blot and quantitative analysis of Fnta protein expression in AMCMs following stimulation with the indicated lipids or glucose (n=3/group); **(d)** Western blot analysis and quantification of Srebf2 protein expression in AMCMs under various metabolic stress conditions (n=4/group). All data represent mean ± SEM. Statistical significance was determined by unpaired two-tailed Student’s t-test for two-group comparisons, or two-way ANOVA followed by Tukey-Kramer post hoc test for multi-group comparisons. ns: not significant.


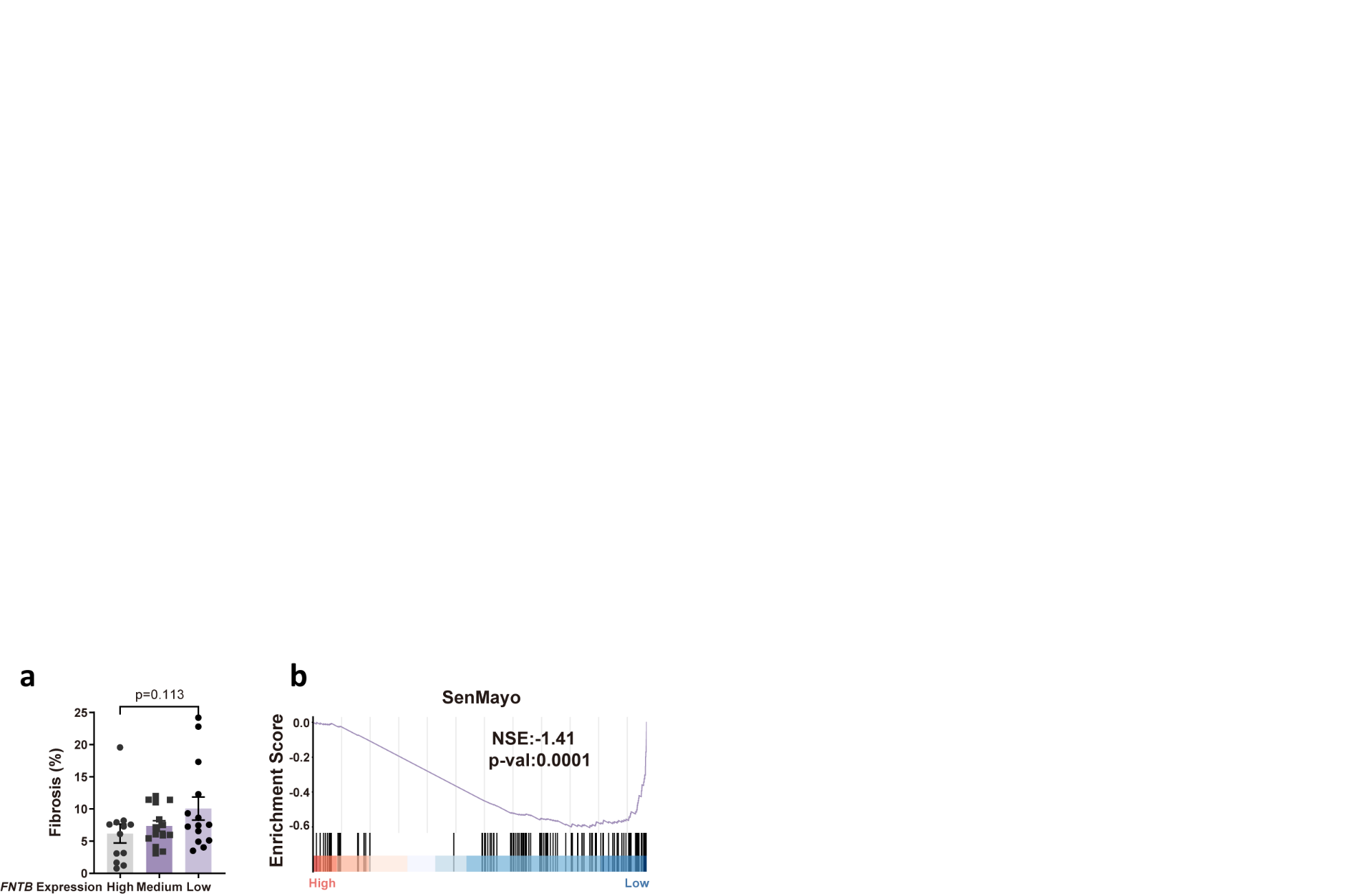


**Figure S13. Association of *FNTB* Expression with Cardiac Fibrosis and Cellular Senescence in heart failure with preserved ejection fraction (HFpEF) Patients.**

We analyzed publicly available RNA-seq data from heart tissues of patients with HFpEF. **(a)** Evaluation of cardiac fibrosis levels across different FNTB expression groups. HFpEF samples were stratified into low, medium, and high FNTB expression groups based on tertiles (0–33%, 33–66%, and 66–100%). Fibrosis scores were obtained from the original study metadata. **(b)** Gene set enrichment analysis (GSEA) comparing the transcriptomic profiles of the FNTB-low group versus the FNTB-high group. The SenMayo gene set, a marker set for cellular senescence, shows significant enrichment in HFpEF hearts with lower FNTB expression. Statistical significance was determined by unpaired two-tailed Student’s t-test for two-group comparisons
